# Supplementary material for: ERAD‐dependent control of the Wnt secretory factor Evi
Source: EMBO J. 2018 Jan 29;37(4):e97311. doi: 10.15252/embj.201797311 (PMC5813261; doi:10.15252/embj.201797311)
Supplement: Supplementary file 1 — Appendix [file EMBJ-37-e97311-s001.pdf]

APPENDIX

TABLE OF CONTENT

Figure S1

Figure S2

Figure S3

Figure S4

Table S1

Table S2

Legends for Appendix Figures and Tables

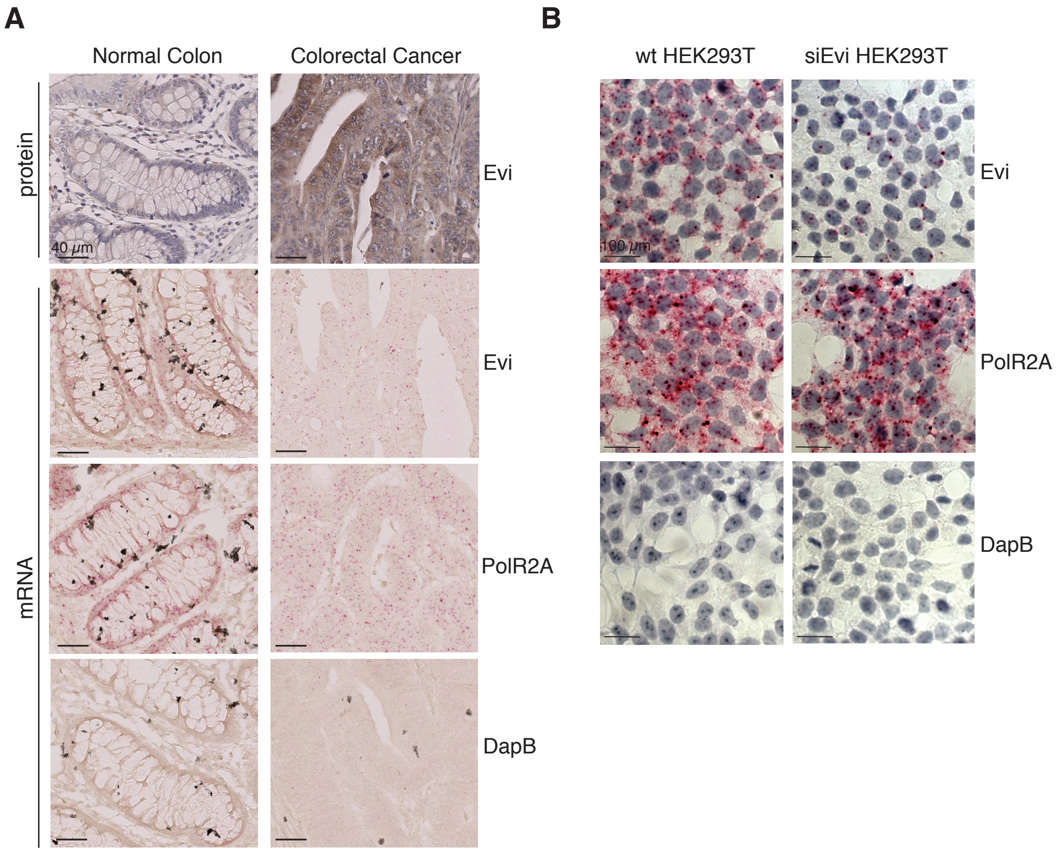

Appendix Figure S1

sgEvi2 #9

```
                Exon 3
Evi^WT      AGTCTCCATGGACGTTTCCCTGGCTTACCGTGATGACGCGT    wt
Evi^KO2.9   AGTCTCCATGGA-GTTTCCCTGGCTTACCGTGATGACGCGT    -1
            AGTCTCCATG-------------------------ACGCGT    -25
            AGTCTCCAT--ACGTTTCCCTGGCTTACCGTGATGACGCGT    -2
```

sgEvi1 #1

```
                Exon 2              Intron 2
Evi^WT      TAAACAACCAAAT-CAGTTAAGTGTACTCTCCTCTCATCCCTTT    wt
Evi^KO1.1   TAAACAACCAAAT----------GTACTCTCCTCTCATCCCTTT    -9 (-2 in Exon)
            TAAACAACCAA---CAGTTAAGTGTACTCTCCTCTCATCCCTTT    -2
            TAAACAACCAAATGCAGTTAAGTGTACTCTCCTCTCATCCCTTT    +1
            TAAACAACCAAAT----TTAAGTGTACTCTCCTCTCATCCCTTT    -3 (-2 in Exon)
            TAAACAACCAAA-----TTAAGTGTACTCTCCTCTCATCCCTTT    -4 (-3 in Exon)
```

Appendix Figure S2

sgPorcn1 #2

Exon 2

$Porcn^{WT}$ TCCTGCCTACTGCCCAGCAGGGCCTTGACCAGATCTGGCTGCTCC wt

$Porcn^{KO1.2}$ TCCTGCCTACT-----------------CCAGATCTGGCTGCTCC -17

sgPorcn1 #4

Exon 2

$Porcn^{WT}$ TCCTGCCTACTGCCCAGCAGGGCCTTGACCAGATCTGGCTGCTCC wt

$Porcn^{KO1.4}$

TCCTGCCTACTGCCCAGCAGGGCCTT--CCAGATCTGGCTGCTCC -2

TCCTGCCTACTGCCCAGCAGGGCCTT-----GATCTGGCTGCTCC -5

TCCTGCCTACTGCCCAGCAGGGCCTTG-CCAGATCTGGCTGCTCC -1

TCCTGCCTACTGCCCAGCAGGGCCTTG-CCCGATCTGGCTGCTCC -1

Appendix Figure S3

A

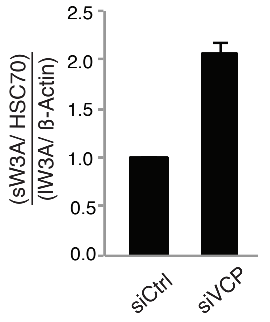

B

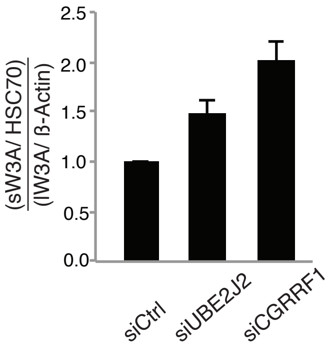

Appendix Figure S4

Appendix Table S1: Oligonucleotide sequences

qRT-PCR Primer

Primer name	Roche Probe	Sequence (5'-> 3')
EVI_fwd	#60	ATGGAGAAGACCAGTCCAATG
EVI_rev	#60	GATAGTGGTGGTGAGCTGGAG
VCP_fwd	#35	AGAGGCAGACAAACCCATCA
VCP_rev	#35	AGTGATCTCGACGGATCTCAG
AXIN2_fwd	#88	AGAGCAGCTCAGCAAAAAGG
AXIN2_rev	#88	CCTTCATACATCGGGAGCAC
GAPDH_fwd	#60	AGCCACATCGCTCAGACAC
GAPDH_rev	#60	GCCCAATACGACCAAATCC
GP78_fwd	#69	GTGCAGCGTAAGGACGAACT
GP78_rev	#69	AGAGGACGCACCTTCCAA
TRC8_fwd	#50	caaagccggttctgcatc
TRC8_rev	#50	actggatgcaaatacaccaaag
CGRRF1_fwd	#46	gggctctacccagagcaag
CGRRF1_rev	#46	agaaaagcggcgagtattca
RNF5_fwd	#78	atgcccatgagcctttcc
RNF5_rev	#78	cgagaaacaggaagagggaat
MARCH6_fwd	#88	ggaggaagatgacgctggt
MARCH6_rev	#88	gcattccaattcatgtcatcc
RNF170_fwd	#62	aacagcttcaaacagaacagga
RNF170_rev	#62	tgtcagtgtagaactgctgtcg
HRD1_fwd	#79	tgaggaccgtgtggacttta
HRD1_rev	#79	gacgaagaggaagtccaggat
RNF128_fwd	#86	ggaacacgtgcagtcaacaa
RNF128_rev	#86	tgggttgtcaacatgaggaa
UBE2J1_fwd	#73	tgctacggccagtacatcg
UBE2J1_rev	#73	acaggttgggtaggttgatga
UBE2J2_fwd	#22	tcctgactgggctcctga
UBE2J2_rev	#22	tgcactgccagttgtctttt

guide RNA

sgEVI2	TGGACGTTTCCCTGGCTTAC
sgEVI1	AATCAGTTAAGTGTACTCTC
sgPORCN	TGACCAGATCTGGCTGCTCC

Amplicon Sequencing Primer

**for First PCR**	*Adapter sequence; Gene specific sequence*
sgEVI_ex3_fwd	TCCCTACACGACGctcttccgatctGCGATCTGGGTGTCCATAGT
sgEVI_ex3_rev	AGTTCAGACGTGTGctcttccgatctAGCTACCGTCAGAAGGCAAA
sgEVI_ex2_fwd	TCCCTACACGACGctcttccgatctATGCCCGTAAGAACCATCAC
sgEVI_ex2_rev	AGTTCAGACGTGTGctcttccgatctGGGAAGAAAGGGATGAGAGG

sgPORCN_fwd1	TCCCTACACGACGctcttccgatctCTCTGTGTCCCTGCTTTGAC
sgPORCN_rev1	AGTTCAGACGTGTGctcttccgatctTGTGGGAGGTCTGATATGGC
sgPORCN_fwd2	TCCCTACACGACGctcttccgatctGTCCCTGCTTTGACAGATCT
sgPORCN_rev2	AGTTCAGACGTGTGctcttccgatctGGAGGTCTGATATGGCCTGG
**for Second PCR**	*Library specific barcode*
Sq_Lib_fwd	AATGATACGGCGACCACCGAGATCTACAC-XXXXXXXX-ACACTCTTTCCCTACACGACGCTCTTCCGATCT
Sq_Lib_rev	CAAGCAGAAGACGGCATACGAGAT-XXXXXXXX-GTGACTGGAGTTCAGACGTGTGCTCTTCCGATC

Cloning Primer

UBE2J2-V5_rev	GGATAGGCTTACCTTCGAAGGCCTCCTGCGCGATGCTCCTCAGCAC
UBE2J2-V5_fwd	TATAGGGAGACCCAAGCTTGGTACGATGAGCAGCACCAGCAGTAAGAGGGCTCC
Wnt3A-KDEL_fwd	CGTGCACACCTGCAAGAAAGATGAGTTGTGAGGGCGCGCCG ACCAGC
Wnt3A-KDEL_rev	GCTGGTCGGCGCGCCCTCACAACTCATCTTTCTTGCAGGTGTGCACG
CGRRF1-C2/3A_fwd	CTGTGTTGTTGCCCAGAATGGGACTGTGAACTGGGTACTCTTACCAGCCAGACACACATGC
CGRRF1-C2/3A_rev	GCATGTGTGTCTGGCTGGTAAGAGTACCCAGTTCACAGTCCCATTCTGGGCAACAACACAG
CGRRF1-C2/4A_fwd	GACTGTGTTGTTGCCCAGAATGGGACTGTGAACTGGGTACTCTTACCATGCAGACACACAGCCCTGTGTGATG
CGRRF1-C2/4A_rev	CATCACACAGGGCTGTGTGTCTGCATGGTAAGAGTACCCAGTTCACAGTCCCATTCTGGGCAACAACACAGTC

Appendix Table S2: siRNAs

Gene Name	Dharmacon Cat No.	Target Sequence
siCtrl non-targeting#1	D-001210-01	UAGCGACUAAACACAUCAA
siCtrl non-targeting#2	D-001210-02	UAAGGCUAUGAAGAGAUAC
siCtrl non-targeting#3	D-001210-03	AUGUAUUGGCCUGUAUUAG
siCtrl non-targeting#4	D-001210-04	AUGAACGUGAAUUGCUCAA
siUBC#1	D-019408-01	GUGAAGACCCUGACUGGUA
siUBC#2	D-019408-02	AAGCAAAGAUCCAGGACAA
siUBC#3	D-019408-03	GAAGAUGGACGCACCCUGU
siUBC#4	D-019408-04	GUAAGACCAUCACUCUCGA
siUBE2J2#1	D-008614-01	GAAGGUGGCUAUUAUCAUG
siUBE2J2#2	D-008614-02	GCACAAGACGAACUCAGUA
siUBE2J2#4	D-008614-04	GUAUAGAGACGUCGGACUU
siUBE2J2#18	D-008614-18	CCCAGUAUCUAUAUGAUCA
siUBE2G2#1	D-009095-01	UCUAUAAGAUUGCCAAGCA
siUBE2G2#2	D-009095-02	GAUGGGAGAGUCUGCAUUU
siUBE2G2#3	D-009095-03	CCACUUGAUUACCCGUUAA
siUBE2G2#5	D-009095-05	GAGCUAACGUGGAUGCGUC
siUBE2J1#1	D-007266-01	GAAAGAAGCGGCAGAAUUG
siUBE2J1#3	D-007266-03	GAAUAUAUCUGGCAAACGA
siUBE2J1#19	D-007266-19	GGAAGUAUAUGUAAGGUUA
siUBE2J1#20	D-007266-20	GGCUAAUGGUCGAUUUGAA
siUBE2U#1	D-008998-01	GAAGUGGAAUACAAACUAU
siUBE2U#2	D-008998-02	GAAUACUGGUUAAAGAUGA
siUBE2U#3	D-008998-03	GUGAAGAUAUGAUGGAAUG
siUBE2U#4	D-008998-04	CAACCUCAUUUAGUGAUUA
siCGRRF1#1	D-006933-01	GAAGAUAGCCUCCUUACAU
siCGRRF1#2	D-006933-02	GACCUUAGCUGAUGAGGAU
siCGRRF1#3	D-006933-03	UAUGAAUACUCGCCGCUUU
siCGRRF1#4	D-006933-04	CUACAUCGCGGUGGUCUUU
siMARCH4#2	D-023172-02	CAUCACCACUGUGCUUAUA
siMARCH4#3	D-023172-03	GAGCUGGUCAUGAGAGUCA
siMARCH4#5	D-023172-05	GUACUGCUAUGGAUUGUGU
siMARCH4#6	D-023172-06	GAGGAUCGCUACUCACUGG
siRNF128#1	D-007061-01	GAAUUGAGGUGGAUGUUGA
siRNF128#4	D-007061-04	CAAAGAGGCAUACAAGUGA
siRNF128#17	D-007061-17	GGUCAUUGAUCUUCGUUCA
siRNF128#18	D-007061-18	GAGACUGCUGUUCGAGAAA
siRNF5#1	D-006558-01	CGGCAAGAGUGUCCAGUAU
siRNF5#2	D-006558-02	GCUGGGAUCAGCAGAGAGA
siRNF5#3	D-006558-03	GCAAGAGUGUCCAGUAUGU
siRNF5#18	D-006558-18	CCGAAGGGCCAAAUCGCGA
siSYVN1#1 (siHRD1#1)	D-007090-01	CAACAAGGCUGUGUACAUG
siSYVN1#2 (siHRD1#2)	D-007090-02	UGUCUGGCCUUCACCGUUU
siSYVN1#3 (siHRD1#3)	D-007090-03	GGAGAUGCCUGAGGAUGGA
siSYVN1#4 (siHRD1#4)	D-007090-04	CCAAGAGACUGCCCUGCAA
siMARCH6#1	D-006925-01	GAAGACAUAUGUAGAGUGU
siMARCH6#2	D-006925-02	UCAUAGAUCUCGUCGCUUA
siMARCH6#3	D-006925-03	GAAUUGGAAUGCUUUAGAA
siMARCH6#4	D-006925-04	GAGCUUACAUGGGAAAGAA
siRNF139#1 (siTRC8#1)	D-006942-01	GGGAAAAGCUUGACGAUUA
siRNF139#2 (siTRC8#2)	D-006942-02	GAACUGUGCUUAAAAGUAA
siRNF139#4 (siTRC8#4)	D-006942-04	GCACAUGUAUCGAAUUUAC
siRNF139#17 (siTRC8#17)	D-006942-17	AUAAUUAGUGGGUGCGAUU
siRNF170#1	D-007078-01	GAAACUGGAUGAUGAUUCA
siRNF170#2	D-007078-02	GGGCAACCCAGAUCUAUUA
siRNF170#3	D-007078-03	GGCCAAAUAUCAAGGUGAA
siRNF170#4	D-007078-04	GAGAUUGCAUCAGGAUAUU

siAMFR#1 (siGP78#1)	D-006522-01	GCAAGGAUCGAUUUGAAUA
siAMFR#2 (siGP78#2)	D-006522-02	GGAGCUGGCUGUCAACAAU
siAMFR#3 (siGP78#3)	D-006522-03	GAGGACUGCUCAUGUGAUU
siAMFR#4 (siGP78#4)	D-006522-04	CGAGCUGGCUGCCGAGUUU
siVCP#5	D-008727-05	GUAAUCUCUUCGAGGUAUA
siVCP#6	D-008727-06	AAACAGAUCCUAGCCCUUA
siVCP#7	D-008727-07	GAGAGCAACCUUCGUAAAG
siVCP#8	D-008727-08	GCACAGGUGGCAGUGUAUA

APPENDIX FIGURE LEGENDS

Appendix Figure S1

(**A**) *In situ* RNA hybridization and immunohistochemistry of Evi was performed on sequential FFPE slides of healthy colon and matched colon cancer tissue of 5 patients. One representative example for 3 patients is shown. Scale bar: 40 µm. (**B**) Reduced signal of Evi mRNA in HEK293T cells after Evi knockdown confirmed specificity of Evi probes, which were used for *in situ* RNA hybridization. Scale bar: 100 µm. (**A, B**) Probes directed against PolR2A and DapB were used as positive and negative controls respectively.

Appendix Figure S2

Clonal Evi^{KO} HEK293T cells were generated *via* CRISPR/Cas9 using sgRNA #2 targeting the 3rd Exon ($Evi^{KO2.9}$) or sgRNA #1 targeting the Exon 2- Intron 2 intersection of Evi ($Evi^{KO1.1}$). Single cell clones were analyzed by targeted amplicon sequencing. sgRNA target sequences are underlined in the wild-type (wt) sequences and PAM sequences are shown in red. Short lines indicate the count of deletions, which are shown on the right. A blue character specifies an insertion.

Appendix Figure S3

Clonal $Porcn^{KO}$ HEK293T cells were generated using sgRNA #1 targeting the 2nd Exon of Porcn. Single cell clones were analyzed by targeted amplicon sequencing. sgRNA target sequences are underlined in the wild-type (wt) sequences and PAM sequences are shown in red. Short lines indicate the count of deletions, which are shown on the right. A blue character specifies an insertion. Single cell clones were analyzed by targeted amplicon sequencing. sgRNA target sequences are underlined in the wild-type (wt) sequences and PAM sequences are shown in red. Short lines indicate the count of deletions, which are shown on the right. A blue character specifies an insertion.

Appendix Figure S4

(**A, B**) 24 hours after reverse transfection with Ctrl siRNA or pooled siRNAs against VCP, UBE2J2 or CGRRF1, HEK293T cells were transfected with Wnt3A expression plasmids. Using Blue Sepharose, secreted Wnt proteins were precipitated from conditioned medium 24 hours after plasmid transfection. Western blot quantification of secreted Wnt3A relative to HSC70 and relative to the input Wnt3A (divided by β-actin level) (means ± sem, (**A**) n = 3, (**B**) n = 6).

APPENDIX TABLE LEGENDS

Appendix Table S1: Oligonucleotide sequences.

qRT-PCR primer of the indicated oligonucleotide sequences were used with the indicated probes for quantitative PCR applying the Universal Probe Library system. Sequences for guide RNAs were determined using E-CRISP (Heigwer *et al*, 2014). For amplicon sequences, primers of the indicated sequences were used for the 1st and 2nd PCR. Adapter sequences are shown in blue, gene-specific sequences in red and the characters illustrating library specific barcodes in green.

Appendix Table S2: siRNA identifier.

The indicated siRNAs were purchased from Dharmacon and used either as single siRNA or as a pool of four siRNAs targeting the same mRNA.
