# Supplementary material for: ERAD‐dependent control of the Wnt secretory factor Evi
Source: EMBO J. 2018 Jan 29;37(4):e97311. doi: 10.15252/embj.201797311 (PMC5813261; doi:10.15252/embj.201797311)
Supplement: Supplementary file 2 — Expanded View Figures PDF [file EMBJ-37-e97311-s002.pdf]

Expanded View Figures

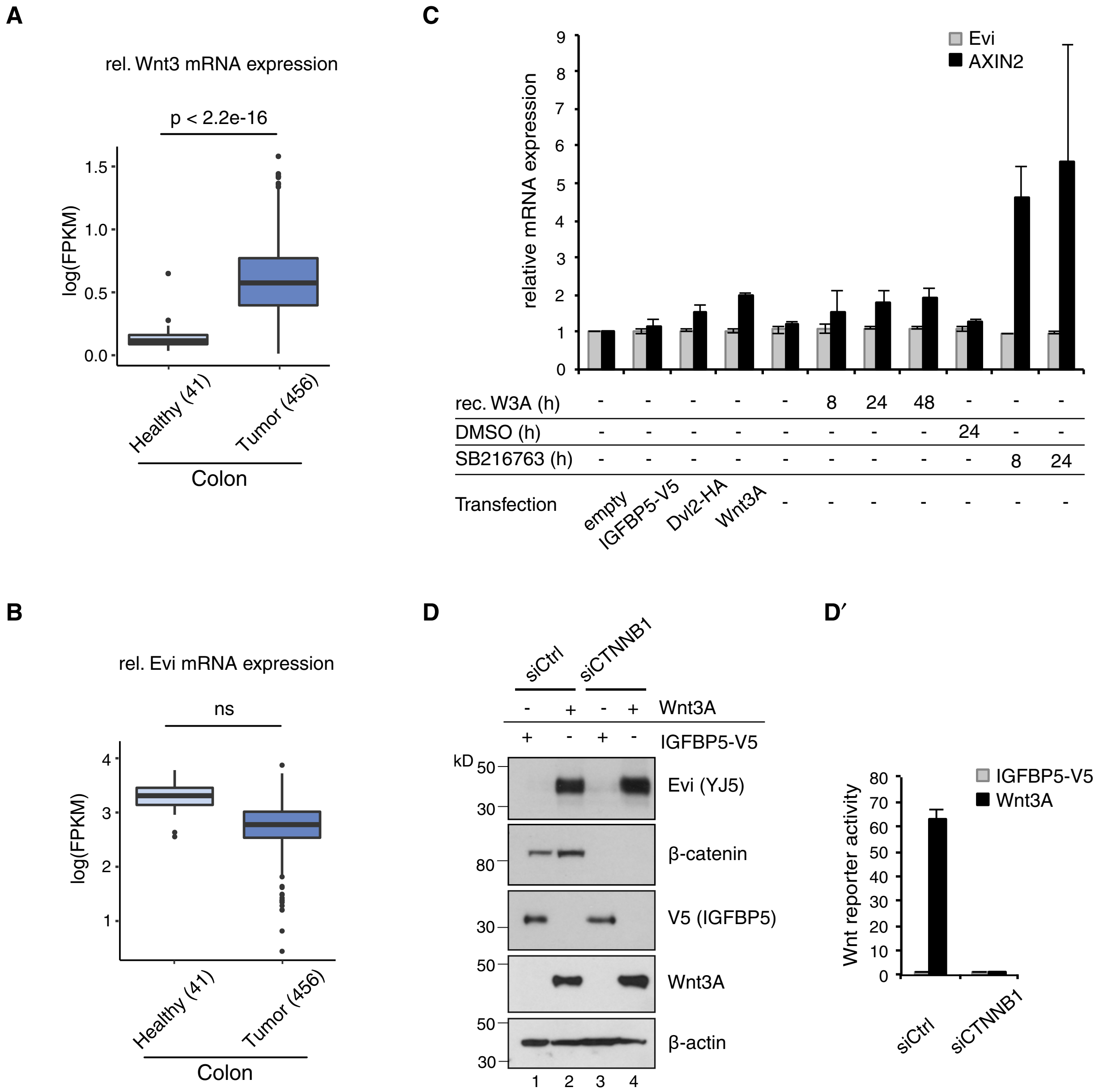

Figure EV1. Evi is not transcriptionally regulated by Wnt.

A, B FPKM-normalized RNA-seq. data of the TCGA Research Network (TCGA-COAD; http://cancergenome.nih.gov/; 09/25/2017) were log-transformed to illustrate the relative expression of (A) Wnt3 and (B) Evi in healthy colon (41) versus colon adenocarcinoma (456). The distribution into tumor and healthy samples was based on their barcodes as described in TCGA Wiki. Statistical significance of gene expression differences was determined using a Student's *t*-test under the alternative hypothesis H_1 that gene expression is higher in tumors compared to healthy tissue. The boxplot diagram shows the median as line within the box, the 25th and 75th percentiles as the upper and lower part of the box, the 10th and 90th percentiles as error bars and outliers as circles.

C HEK293T cells were transfected with the indicated expression constructs, treated with 100 ng/ml recombinant mouse Wnt3A (rec. W3A) or with 10 μM GSK3 inhibitor SB216763 for the indicated hours (h). AXIN2 and Evi mRNA levels were analyzed by qRT–PCR and normalized to GAPDH expression. Results are shown as mean ± s.d. from three independent experiments.

D, D′ Twenty-four hours after reverse transfection with Ctrl or CTNNB1 siRNA, HEK293T cells were transfected with Wnt3A or IGFBP5-V5 expression plasmids and analyzed (D) for the indicated proteins via immunoblotting or (D′) for canonical Wnt activity using the TCF-Luciferase Wnt reporter assay. Immunoblotting is representative of three independent experiments, and Wnt reporter activity was calculated as mean from three independent experiments ± s.d.

Source data are available online for this figure.

© 2018 The Authors

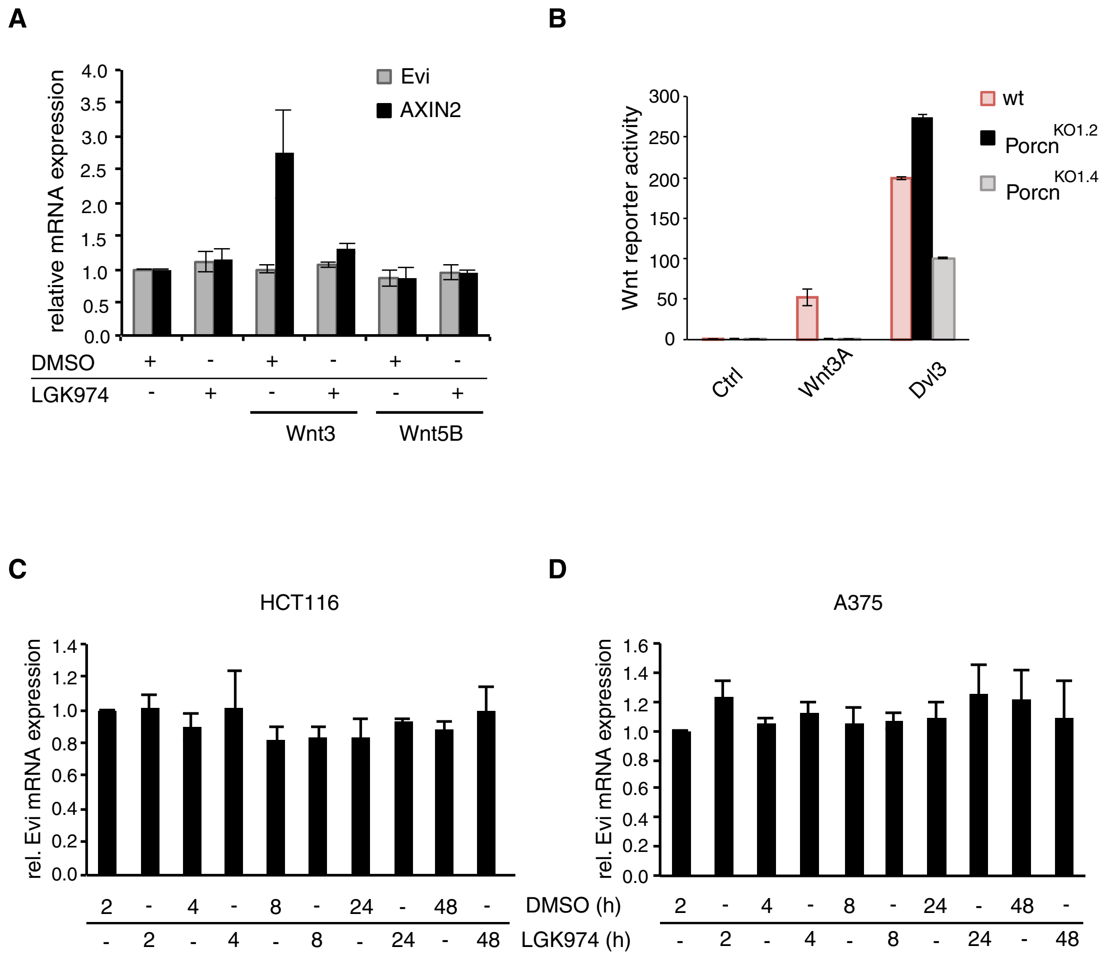

Figure EV2. Porcn inhibition does not affect Evi transcription.

A Wild-type or stable Wnt3- and Wnt5B-expressing HEK293T cells were treated with 5 μM LGK974 for 48 h, and AXIN2 and Evi mRNA levels were quantified by qRT–PCR. Results are shown as mean ± s.d. from three independent experiments.

B Wild-type (wt), Porcn$^{KO1.2}$, and Porcn$^{KO1.4}$ HEK293T cells were transfected with the indicated overexpression constructs and analyzed in Wnt Luciferase activity assays. Results are representative of three independent experiments and shown as mean of seven technical replicates ± s.d.

C, D HCT116 (C) or A375 cells (D) were treated with 5 μM LGK974 for the indicated hours (h). Evi mRNA was quantified by qRT–PCR and normalized to GAPDH. Results are shown as mean ± s.d. from three independent experiments.

Source data are available online for this figure.

Figure EV3. Evi is ubiquitinated and degraded from the ER by the proteasome.

A HEK293T cells were transfected with the indicated expression constructs and treated with Bafilomycin A (Bafilo) in the indicated concentrations for 24 h. Lysosomal inhibition additionally increased Evi protein on top of Wnt3A expression indicating that Evi is degraded by the lysosome also in the presence of Wnt proteins.

B HEK293T cells were transfected with Wnt3A or IGFBP5-V5 expression plasmids and treated for 24 h with DMSO, MG132, or bortezomib in the indicated concentrations.

C Following MG132 treatment (1 μM for 24 h), poly-ubiquitinated proteins were precipitated using TUBE2 agarose and incubated with DUB buffer, supplemented with the catalytic domain of the DUB enzyme USP2, if indicated. The precipitates were assayed for endogenous Evi or K48 poly-ubiquitin. Ctrl agarose beads were used to confirm specificity of the TUBE2 assay for ubiquitinated proteins.

D HEK293T cells were transfected with the indicated overexpression constructs and treated with 5 μM LGK974 for 48 h, if indicated. Secreted Wnt3A proteins were precipitated from conditioned medium and analyzed via immunoblotting. Compared to wild-type Wnt3A, Wnt3A-KDEL is not secreted into the medium affirming cellular retention. HSC70 served as loading control. All Western blots are representative of three independent experiments.

E Upon VCP knockdown in HEK293T cells using single or pooled siRNAs, Evi and VCP mRNA were analyzed via qRT–PCR. mRNA levels are shown as mean ± s.d. relative to GAPDH mRNA levels from three independent experiments.

Source data are available online for this figure.

© 2018 The Authors

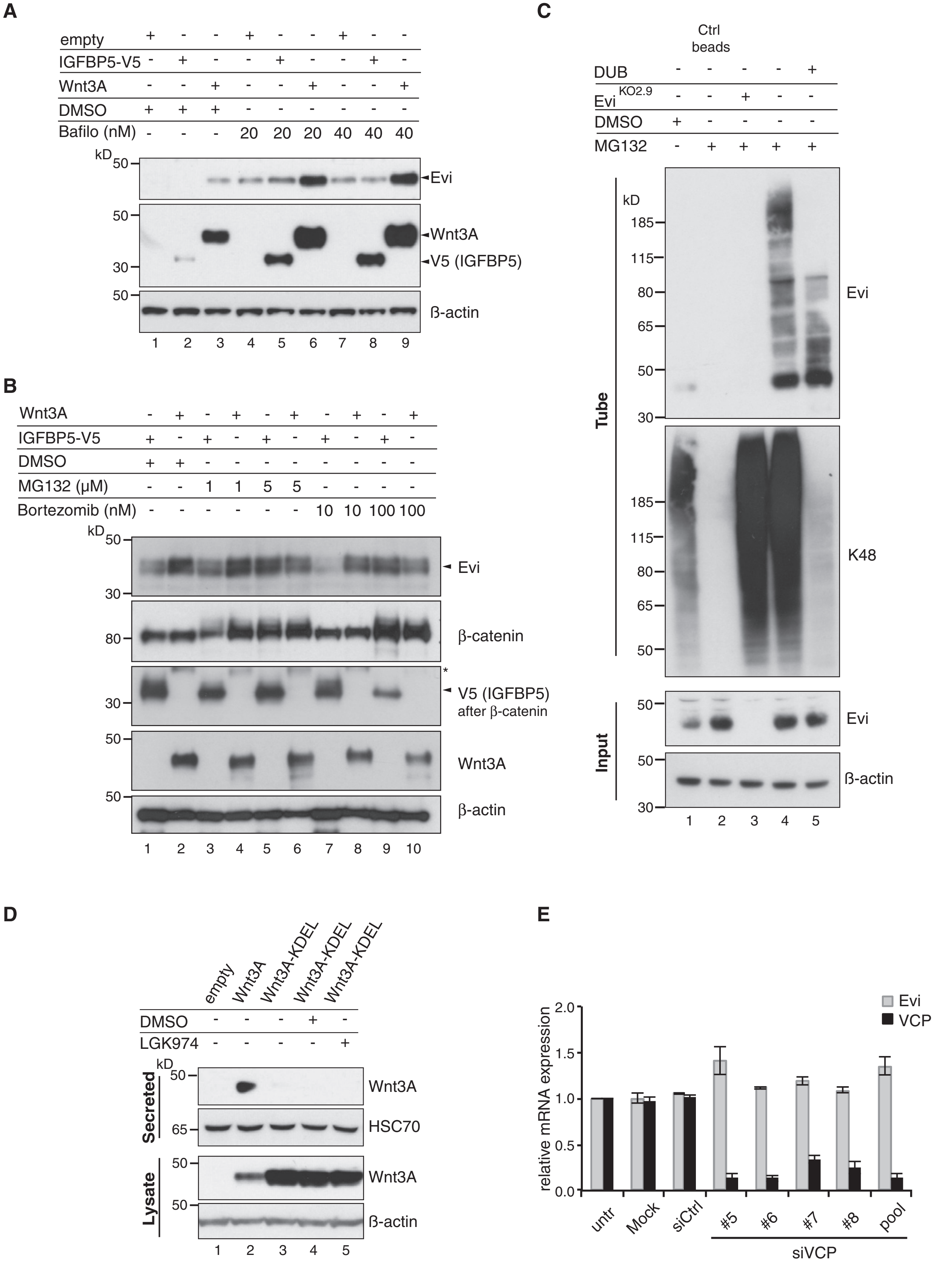

Figure EV3.

 © 2018 The Authors

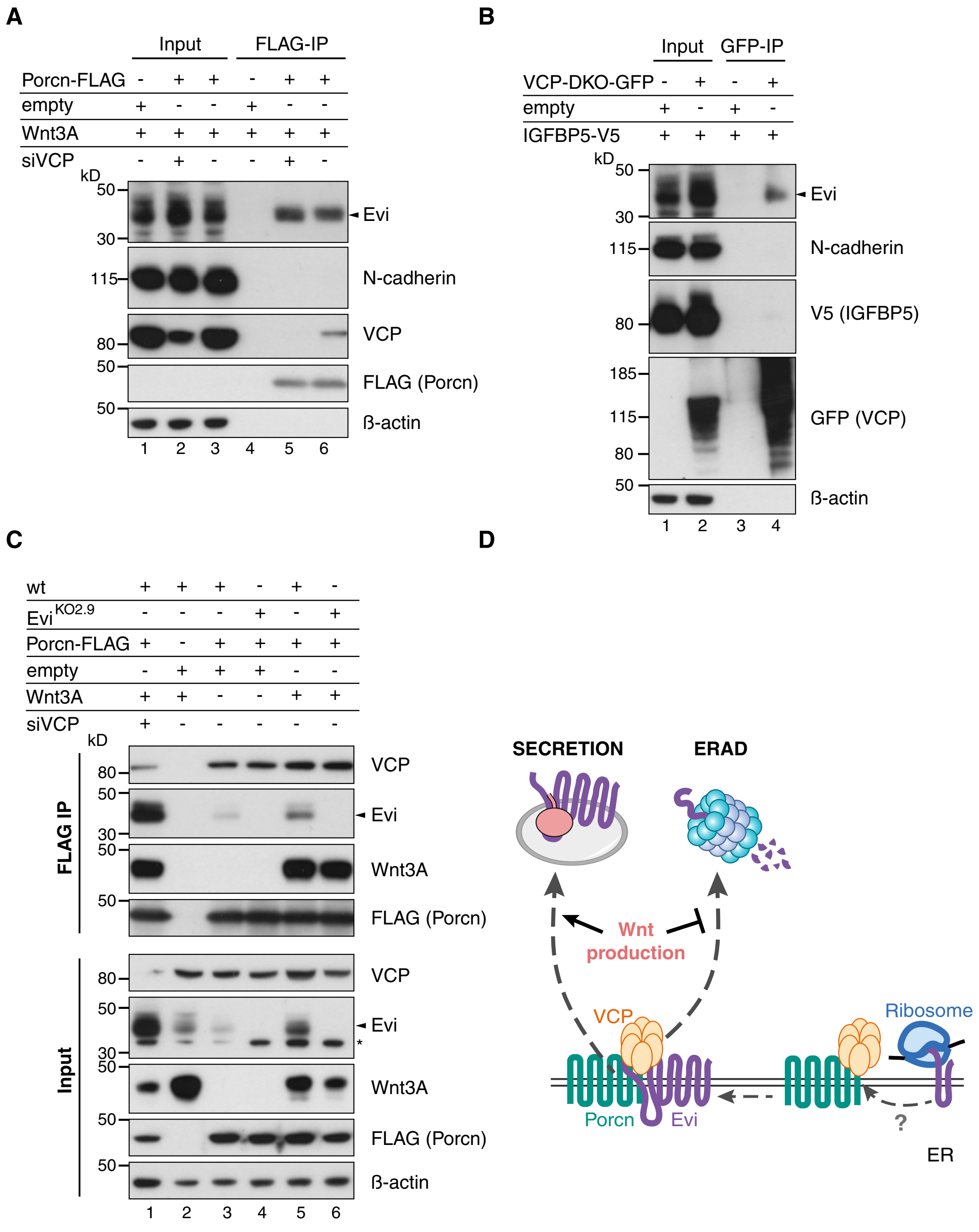

Figure EV4. Specific interaction of Porcn and VCP with endogenous Evi.

A–C Twenty-four hours after reverse transfection with pooled VCP siRNA, wt or Evi$^{KO2.9}$ HEK293T cells were transfected with the indicated overexpression plasmids. Following (A, C) FLAG IP of FLAG-Porcn or (B) GFP IP of GFP-tagged VCP versions, the interacting proteins and the input were analyzed by blotting with the corresponding antibodies. VCP-DKO indicates a catalytically dead mutant form of VCP-containing mutations in both ATPase domains (E305Q/E578Q). Representative Western blots are shown of three (A, C) or one (B, N-cadherin blot) experiments. β-Actin served as loading control.

D Model of a preformed VCP-Porcn complex, which waits for newly translated Evi guiding it either into ERAD or the secretory pathway.

Source data are available online for this figure.

© 2018 The Authors

Figure EV5. The E2 UBE2J2 and the E3 CGRRF1 are involved in Evi regulation.

A Following reverse transfection of HEK293T cells with siCtrl or pooled siRNAs against VCP and the indicated E2 and E3 enzymes, knockdown was confirmed by qRT–PCR. mRNA levels are relative to GAPDH and shown as mean ± s.d. from three independent experiments.

B, C Western blot analysis of endogenous Evi in HEK293T cells upon knockdown of (B) UBE2J2 or (C) CGRRF1 using single or pooled siRNAs. All Western blots are representative of three independent experiments. β-Actin served as loading control.

D, E HEK293T cells were transfected with the indicated overexpression plasmids (FH: FLAG-HA tag) and subjected to FLAG IP. The interacting proteins and the input were analyzed by immunoblotting with the corresponding antibodies. Western blots are representative of three independent experiments.

F Schematic model illustrating the involvement of the E2 conjugating enzyme UBE2J2 and the RING E3 ubiquitin ligase CGRRF1 in conjugating ubiquitin (Ub) to Evi.

G, H FPKM-normalized RNA-seq data of the TCGA Research Network (http://cancergenome.nih.gov/; 09/25/2017) were log-transformed to illustrate the relative expression of (G) CGRRF1 and (H) Evi in healthy colon (41) versus colon adenocarcinoma (TCGA-COAD, 456 samples) and in healthy endometrium (35) versus endometrial carcinoma (TCGA-UCEC; 552 samples). Statistical significance of gene expression differences was determined using a Student's *t*-test under the null hypothesis H_0. The boxplot diagram shows the median as line within the box, the 25th and 75th percentiles as the upper and lower part of the box, the 10th and 90th percentiles as error bars and outliers as circles.

Source data are available online for this figure.

 © 2018 The Authors

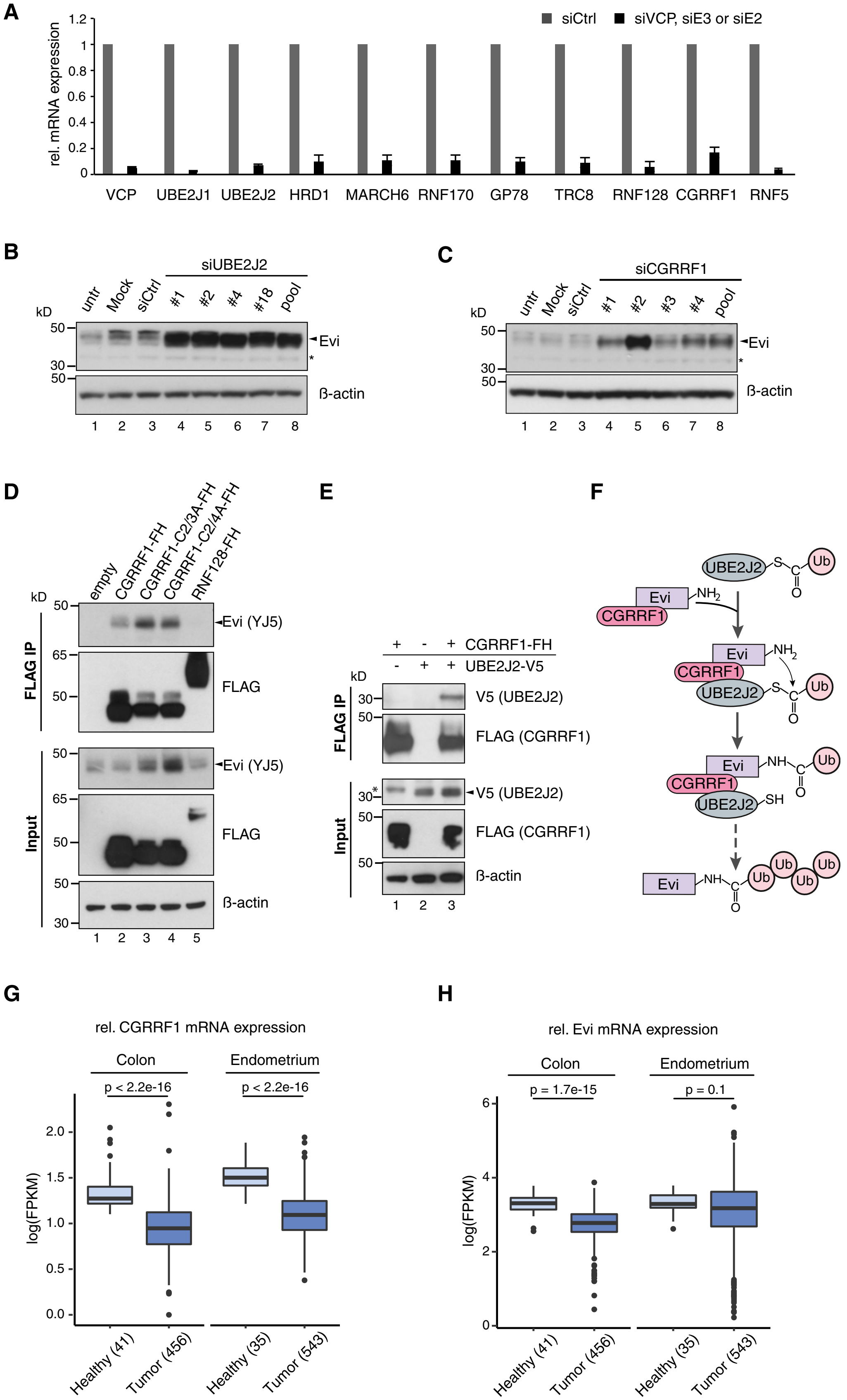
A
siCtrl
siVCP, siE3 or siE2
rel. mRNA expression
VCP
UBE2J1
UBE2J2
HRD1
MARCH6
RNF170
GP78
TRC8
RNF128
CGRRF1
RNF5
B
siUBE2J2
untr
Mock
siCtrl
#1
#2
#4
#18
pool
Evi
ß-actin
C
siCGRRF1
#3
D
empty
CGRRF1-FH
CGRRF1-C2/3A-FH
CGRRF1-C2/4A-FH
RNF128-FH
FLAG IP
Input
Evi (YJ5)
FLAG
E
CGRRF1-FH
UBE2J2-V5
V5 (UBE2J2)
FLAG (CGRRF1)
F
UBE2J2
Ub
Evi
NH2
CGRRF1
SH
G
rel. CGRRF1 mRNA expression
Colon
Endometrium
p < 2.2e-16
log(FPKM)
Healthy (41)
Tumor (456)
Healthy (35)
Tumor (543)
H
rel. Evi mRNA expression
p = 1.7e-15
p = 0.1

Figure EV5.
