# Supplementary material for: ERAD‐dependent control of the Wnt secretory factor Evi
Source: EMBO J. 2018 Jan 29;37(4):e97311. doi: 10.15252/embj.201797311 (PMC5813261; doi:10.15252/embj.201797311)

Fig EV3A

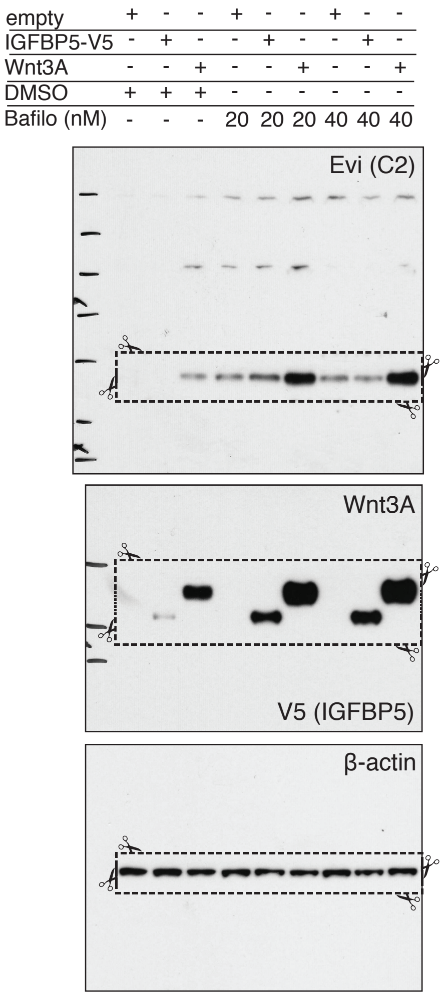


Fig EV3B

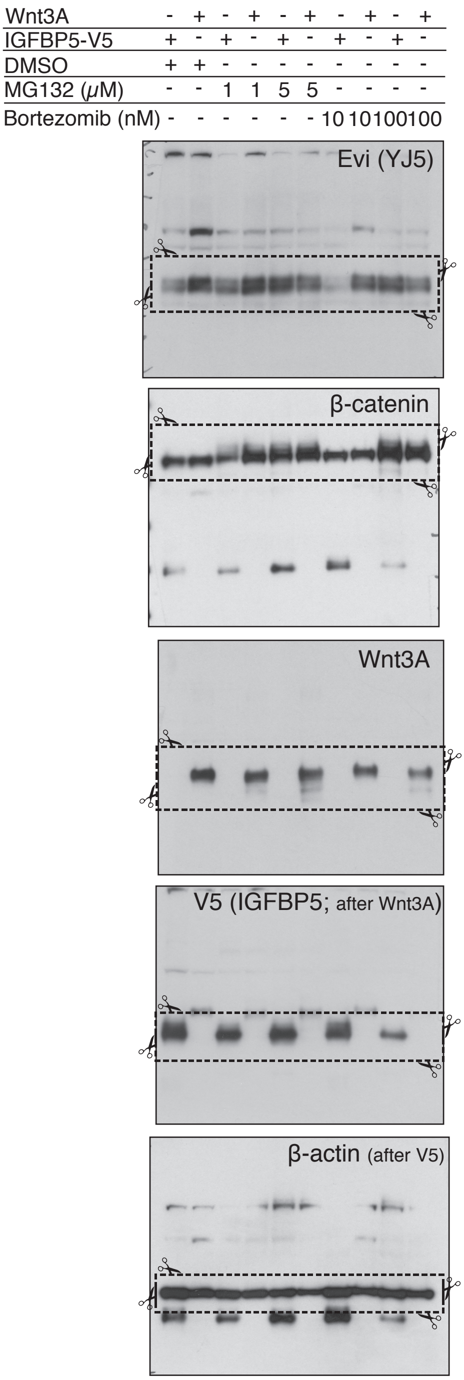

## Fig EV3C

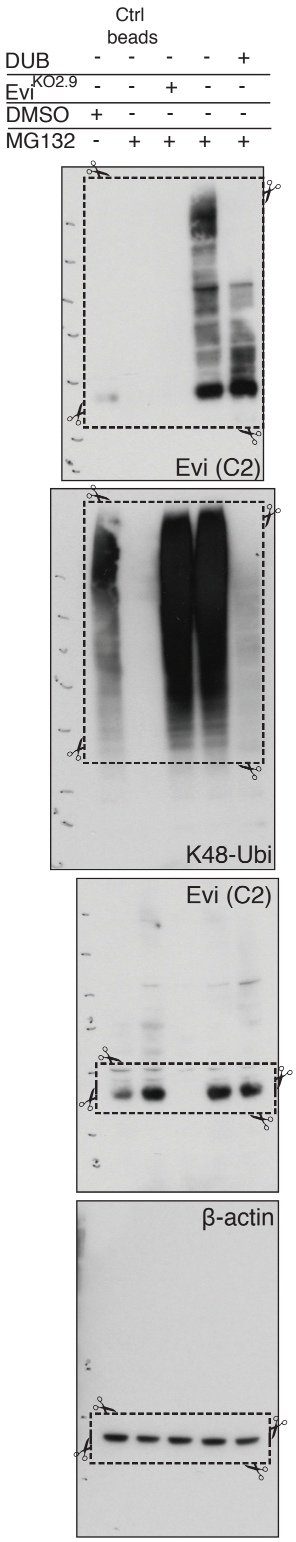


## Fig EV3D

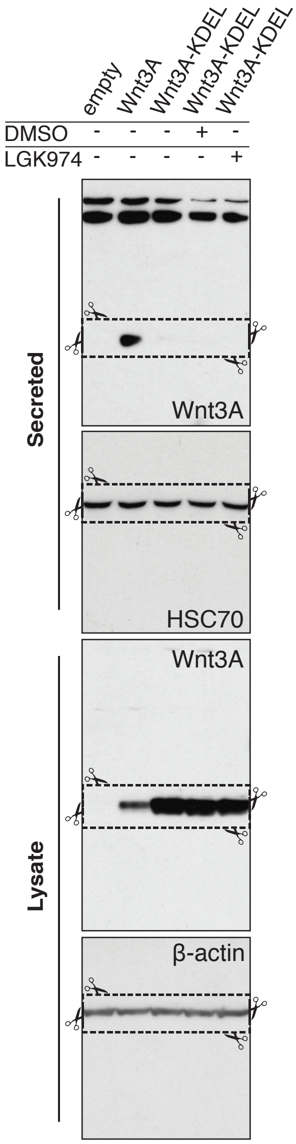

Supplement: Supplementary file 3 — Source Data for Expanded View and Appendix [file EMBJ-37-e97311-s010.zip › Source_Data_EV3/Source_Data_EV3.pdf]
