# Supplementary figures and images for: ERAD‐dependent control of the Wnt secretory factor Evi
Source: EMBO J. 2018 Jan 29;37(4):e97311. doi: 10.15252/embj.201797311 (PMC5813261; doi:10.15252/embj.201797311)

Fig EV4A

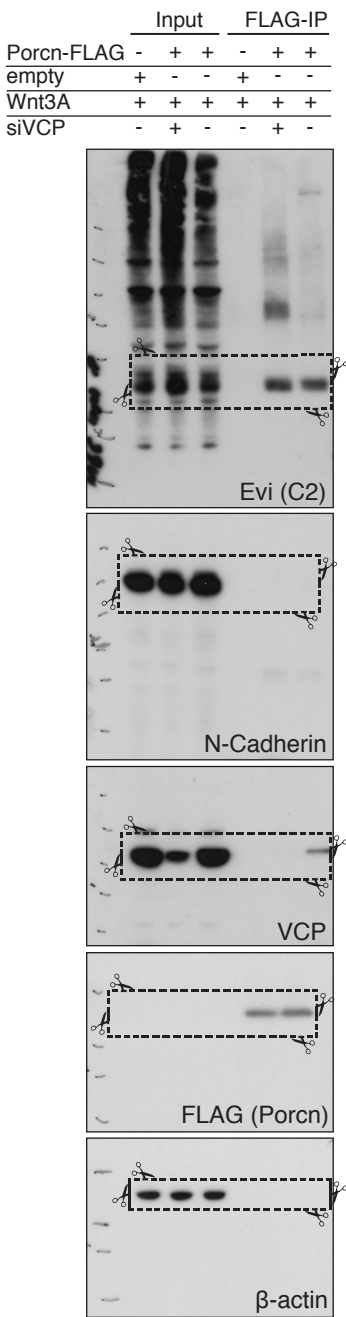

Fig EV4B

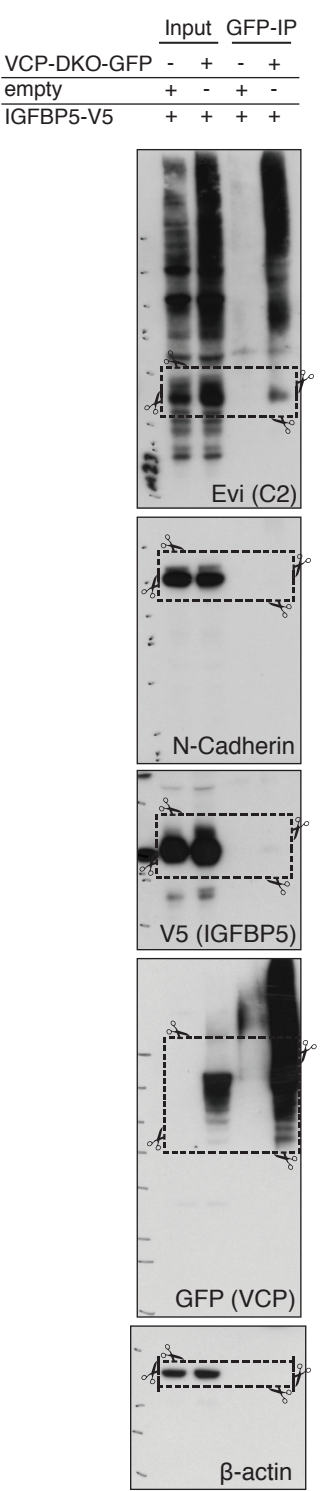

Fig EV4C

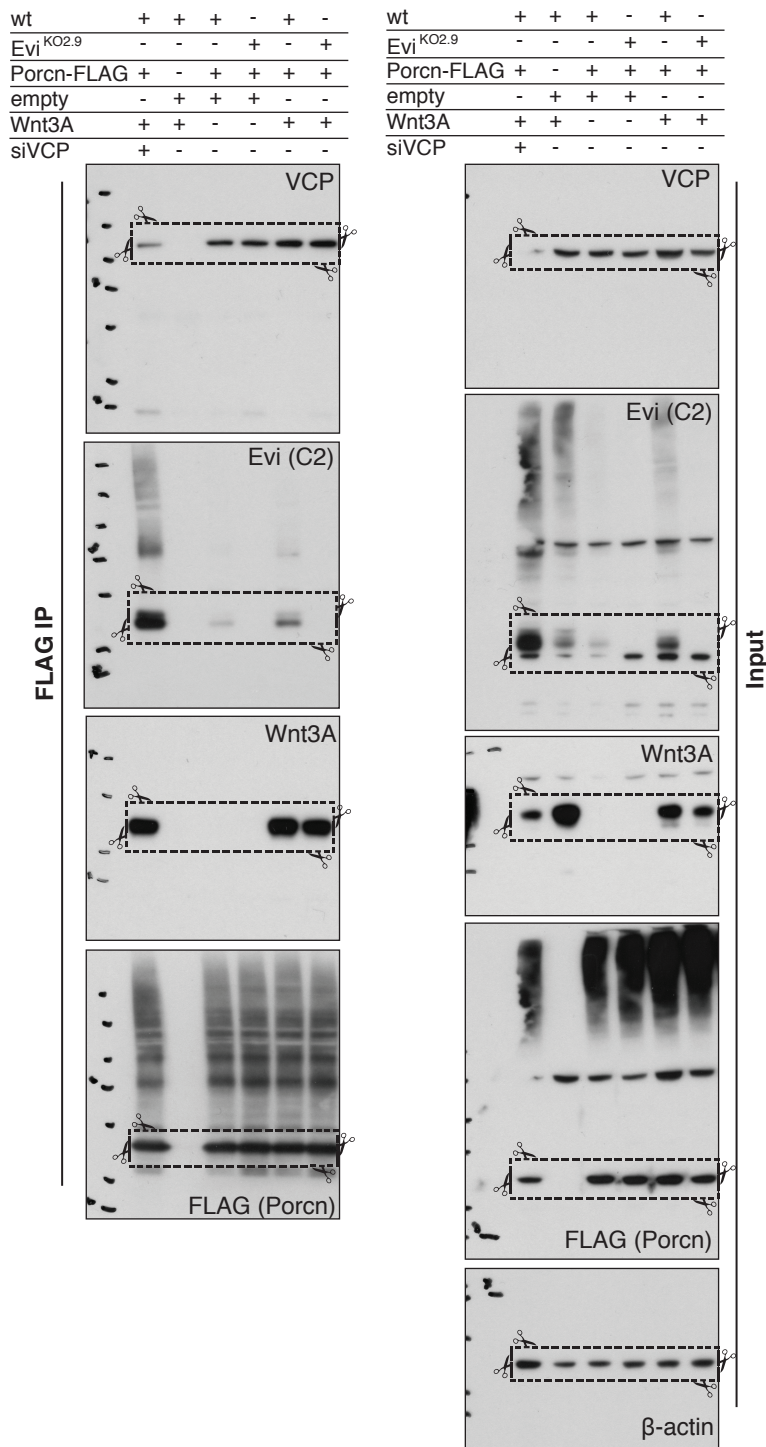

Supplement: Supplementary file 3 — Source Data for Expanded View and Appendix [file EMBJ-37-e97311-s010.zip › Source_Data_EV4.pdf]

Fig EV1D

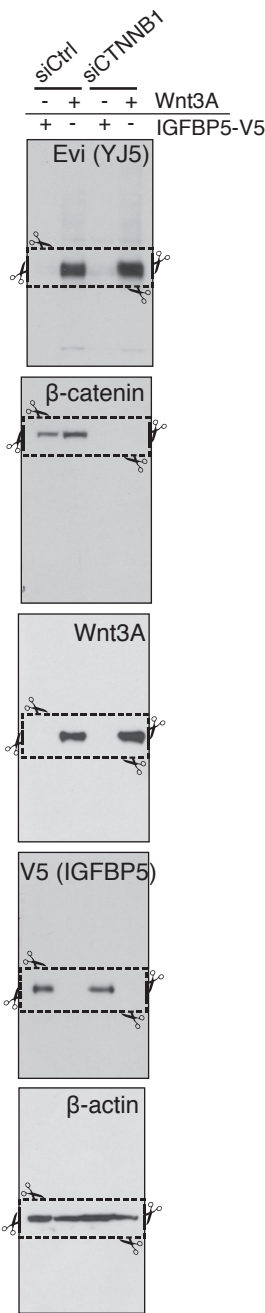

Supplement: Supplementary file 3 — Source Data for Expanded View and Appendix [file EMBJ-37-e97311-s010.zip › Source_Data_EV1/Source_Data_EV1.pdf]

Fig EV5B

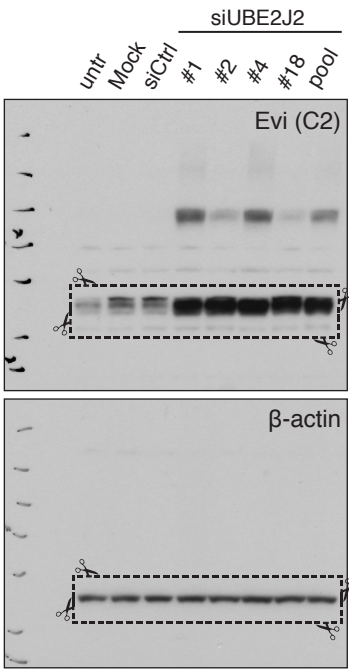

Fig EV5D

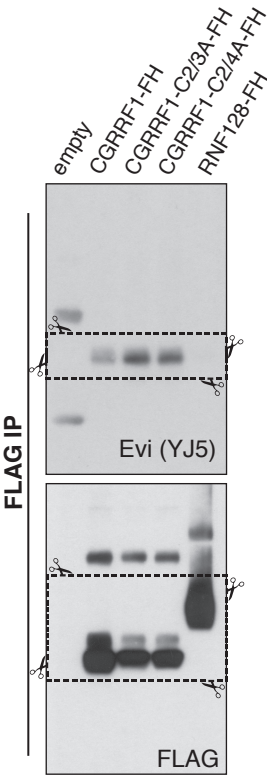

Fig EV5E

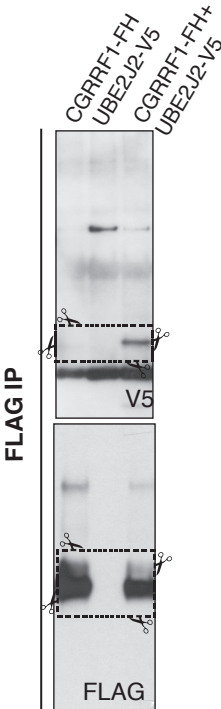

Fig EV5C

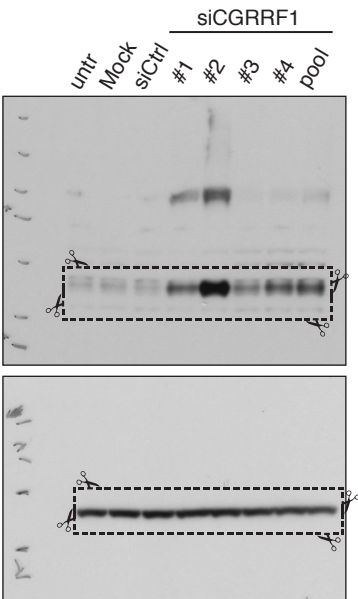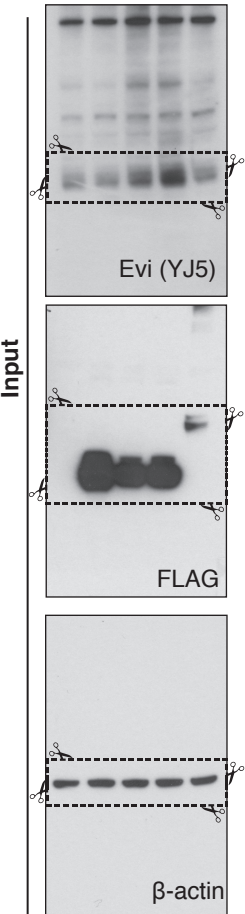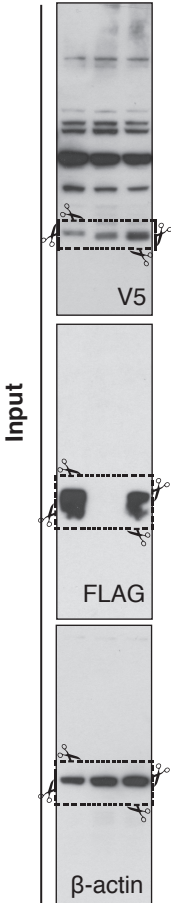

Supplement: Supplementary file 3 — Source Data for Expanded View and Appendix [file EMBJ-37-e97311-s010.zip › Source_Data_EV5/Source_Data_EV5.pdf]

Fig1 B

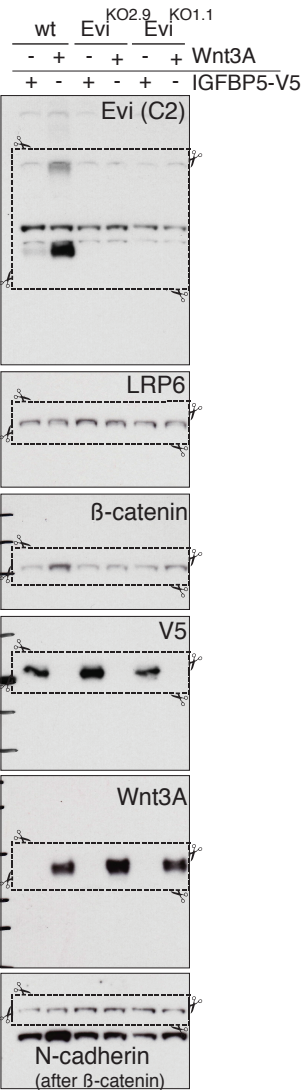

Fig1 C

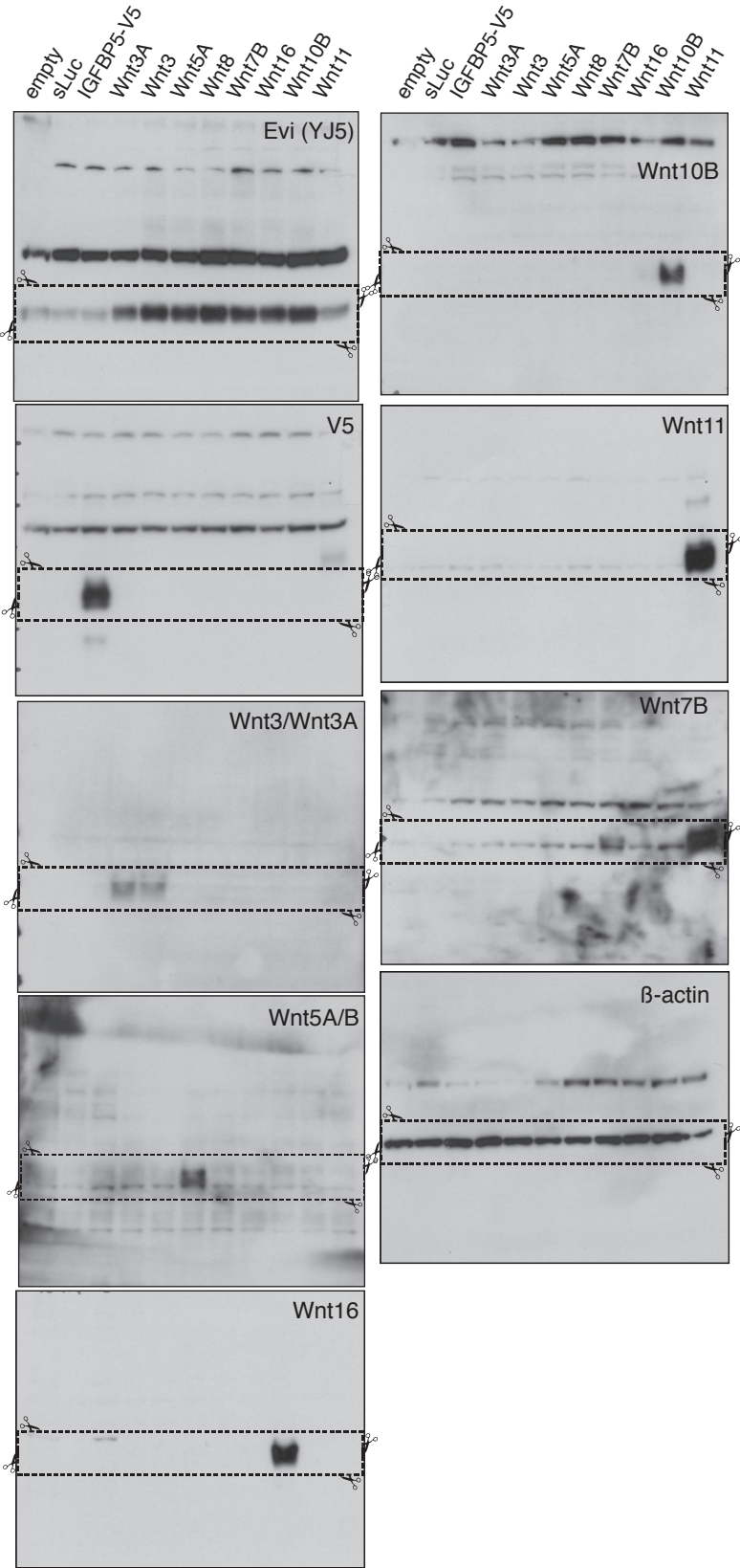

# Fig1D

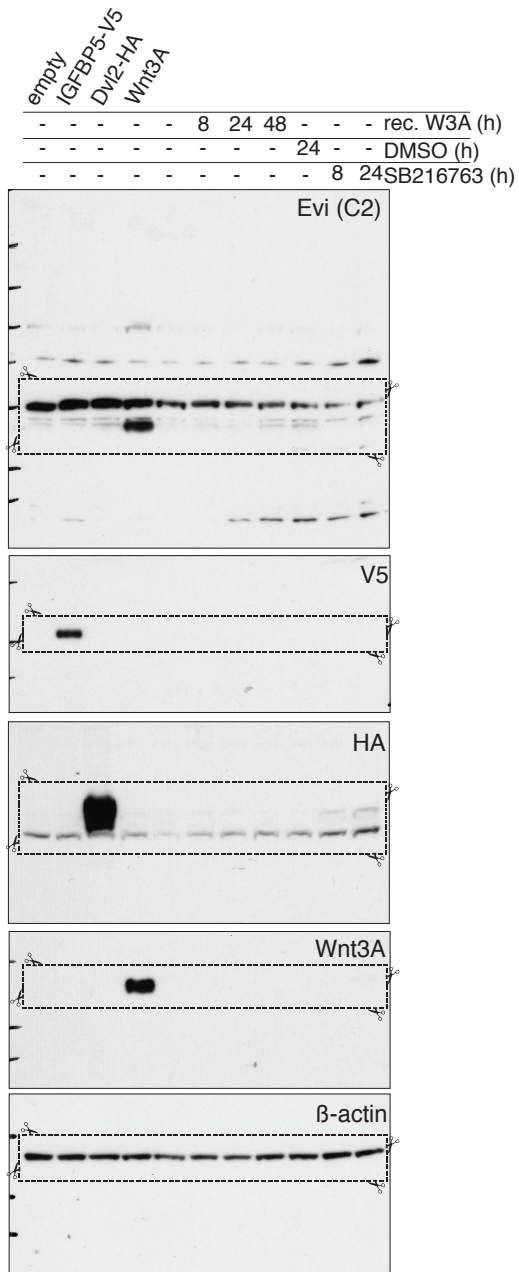

Supplement: Supplementary file 5 — Source Data for Figure 1 [file EMBJ-37-e97311-s003.pdf]

Fig2 B

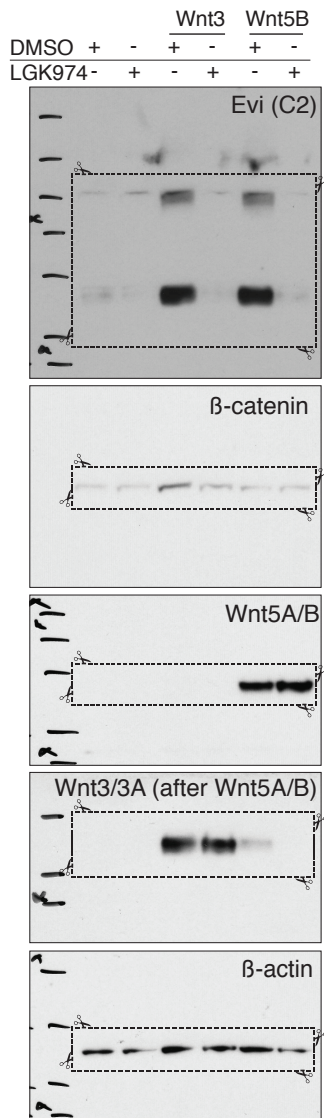

Fig2 C

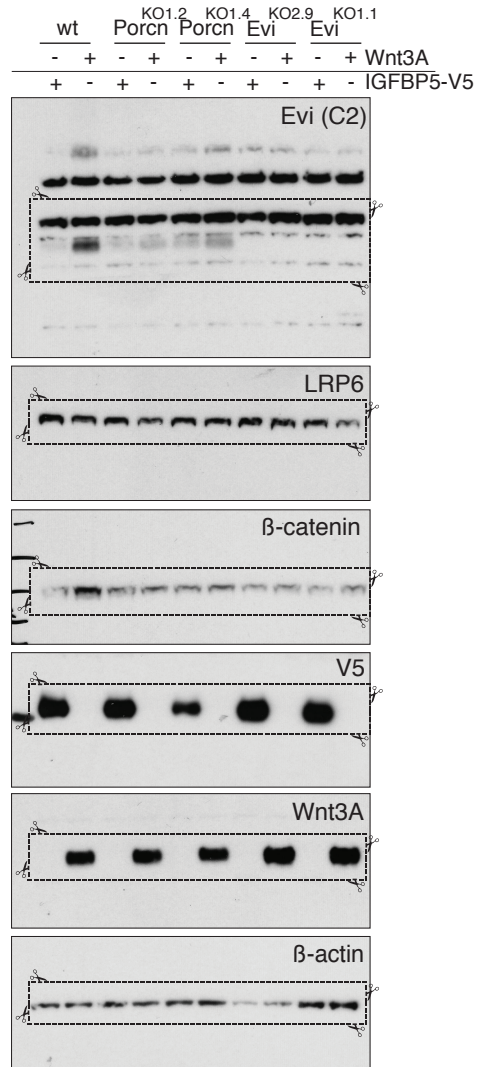

Fig2 D

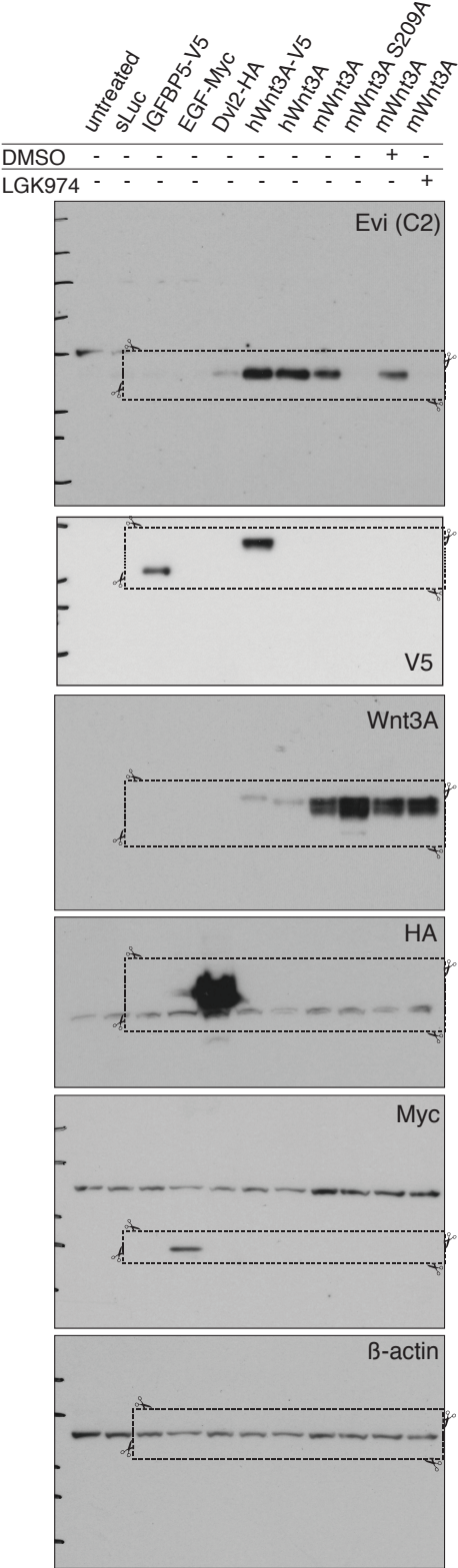

Fig2 E

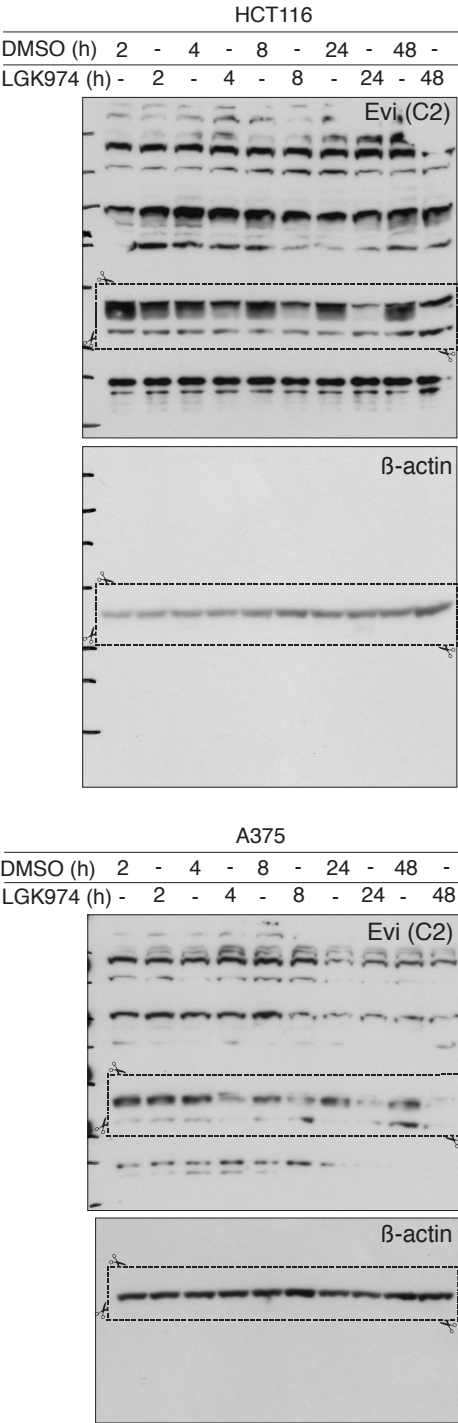

Supplement: Supplementary file 6 — Source Data for Figure 2 [file EMBJ-37-e97311-s004.pdf]

Fig4 B

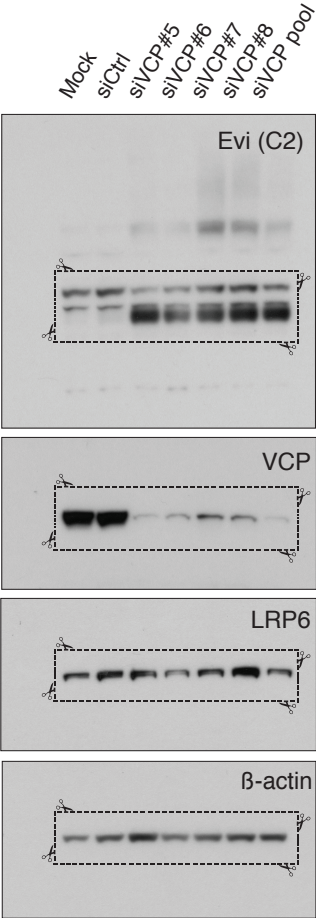

Fig4 C

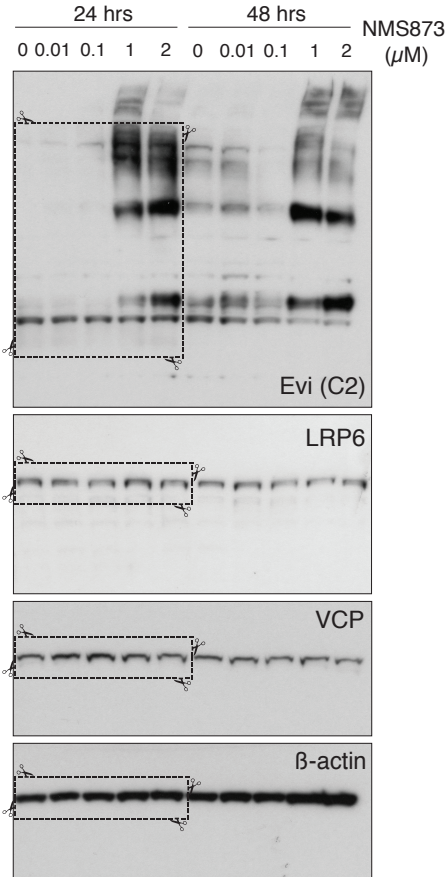

Fig4 D

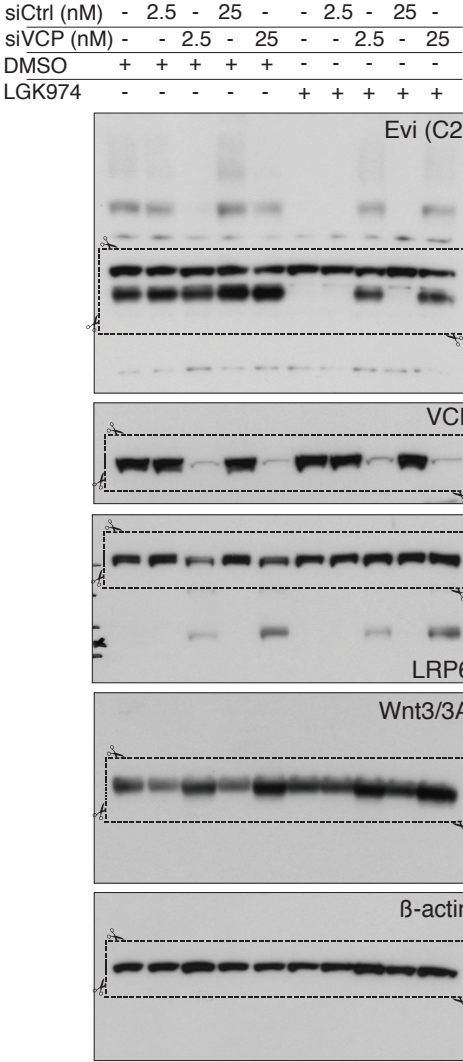

Fig4 E

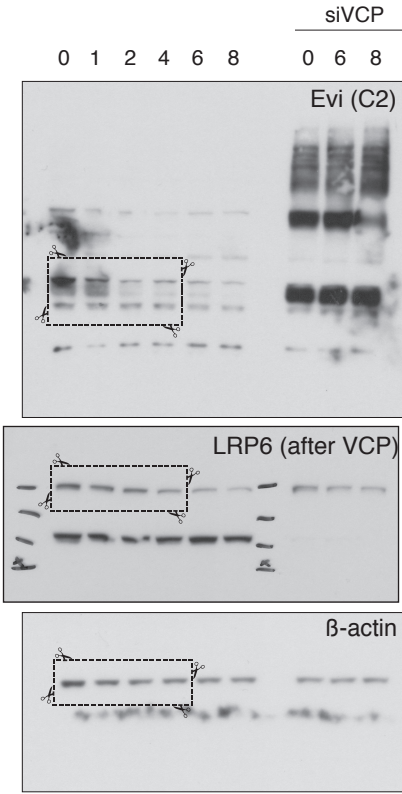

Fig4 E

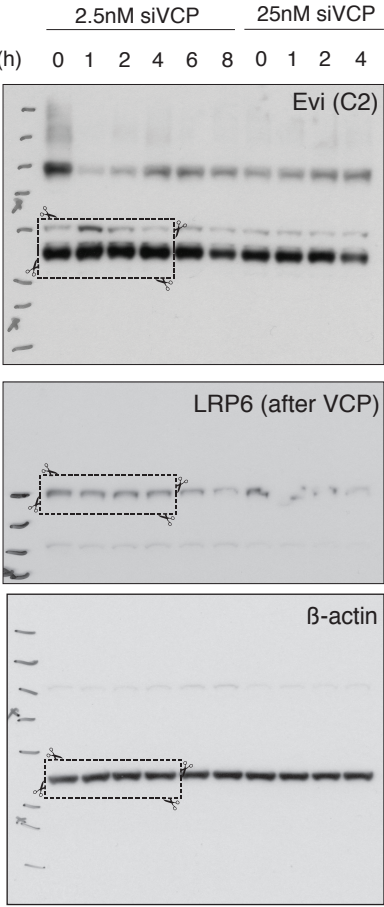

Fig4 F

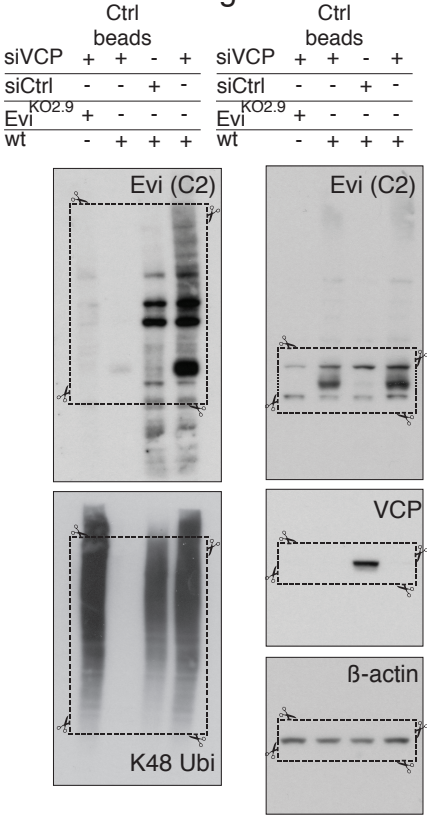

Supplement: Supplementary file 8 — Source Data for Figure 4 [file EMBJ-37-e97311-s006.pdf]

Fig6 B

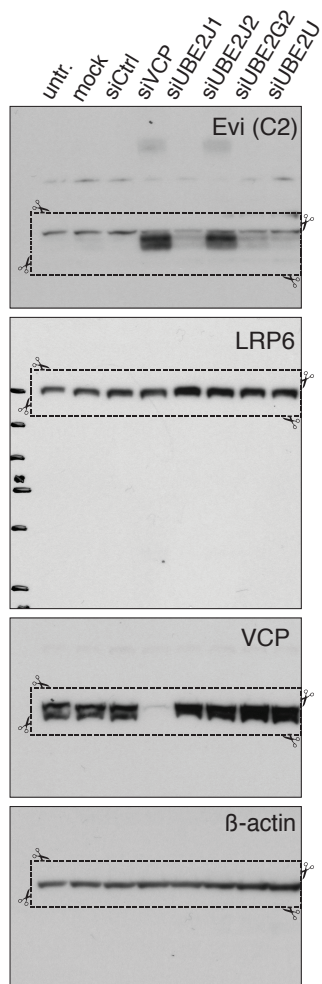

Fig6 C

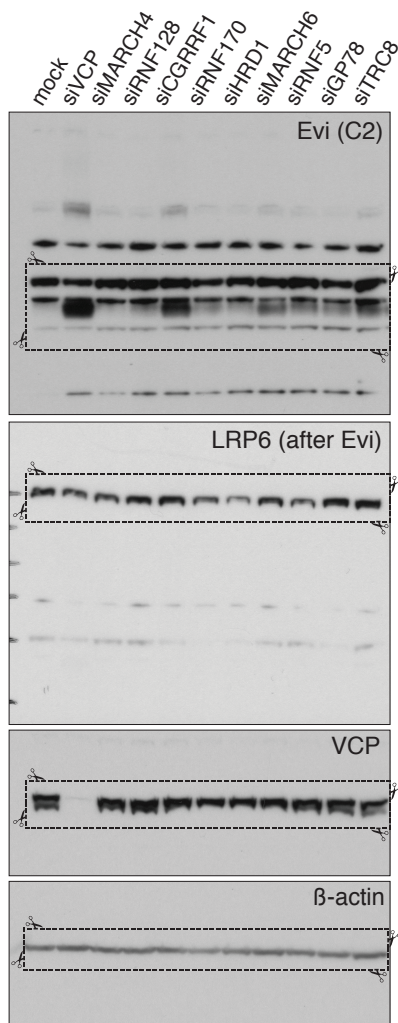

Fig6 D

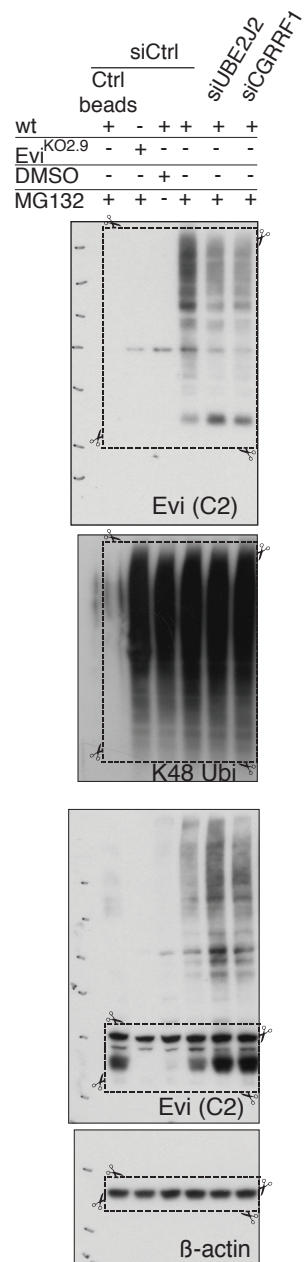

Fig6 E

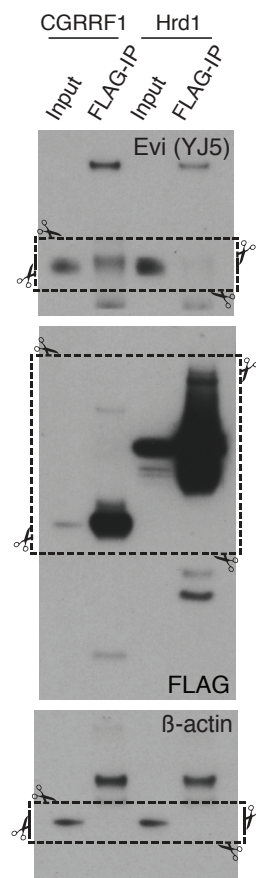

Supplement: Supplementary file 10 — Source Data for Figure 6 [file EMBJ-37-e97311-s008.pdf]

Fig7 A

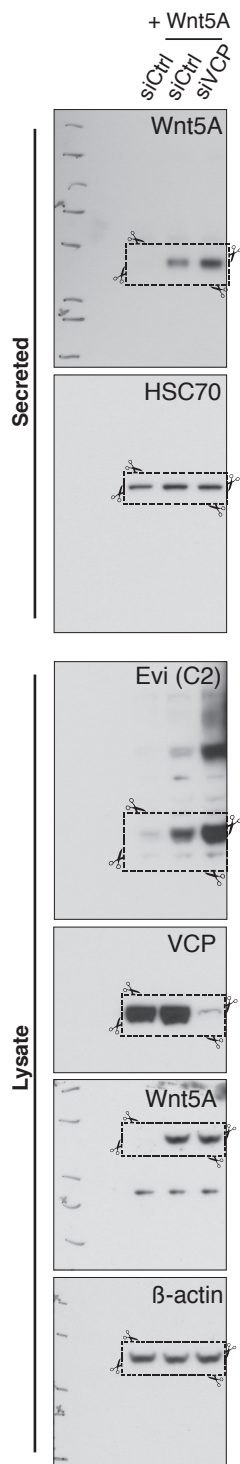

Fig7 B

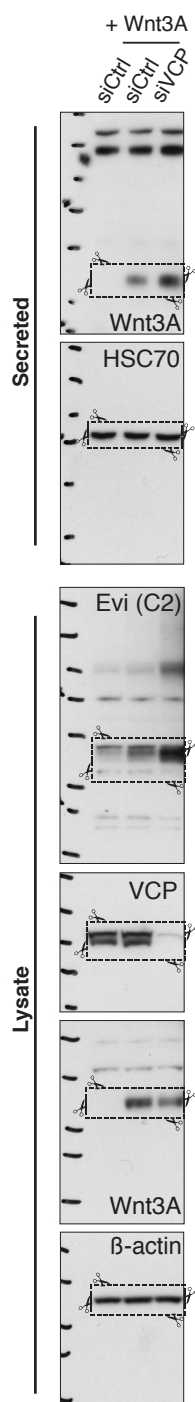

Fig7 C

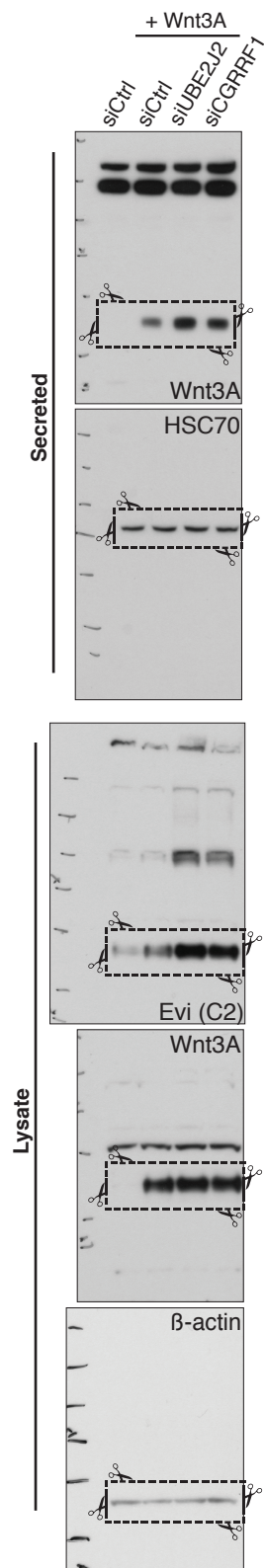

Supplement: Supplementary file 11 — Source Data for Figure 7 [file EMBJ-37-e97311-s009.pdf]
