# Supplementary material for: ERAD‐dependent control of the Wnt secretory factor Evi
Source: EMBO J. 2018 Jan 29;37(4):e97311. doi: 10.15252/embj.201797311 (PMC5813261; doi:10.15252/embj.201797311)
Supplement: Supplementary file 7 — Source Data for Figure 3 [file EMBJ-37-e97311-s005.pdf]

Fig3 A

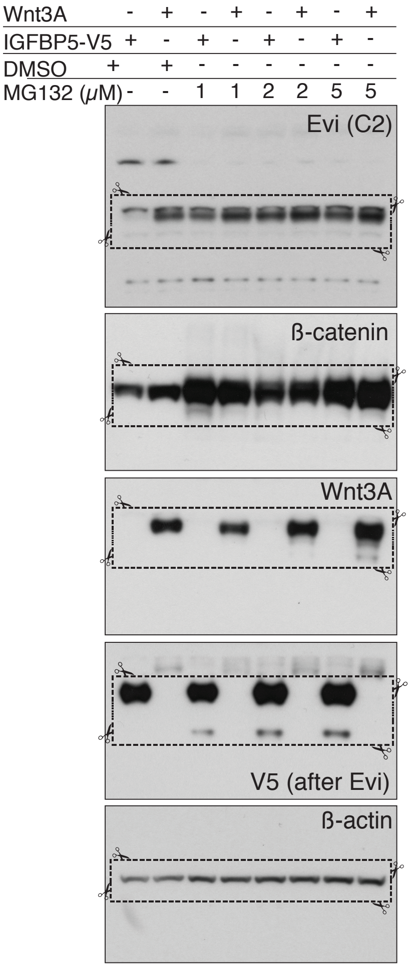

Fig3 B TUBE

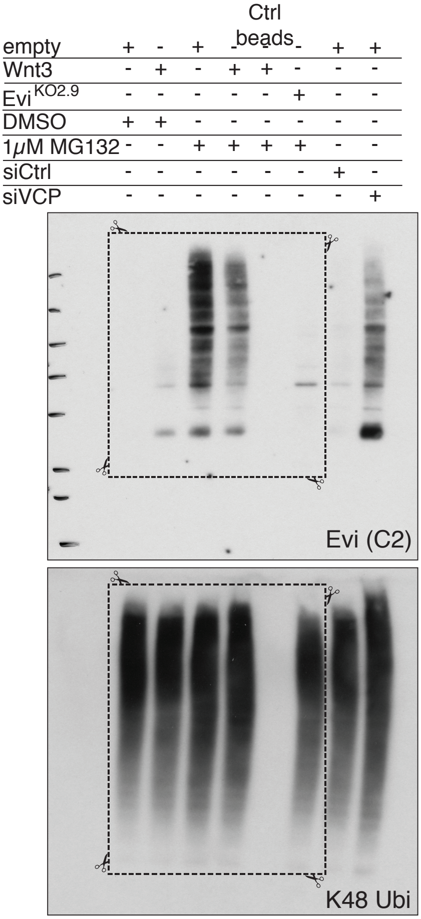

Fig3 B Input

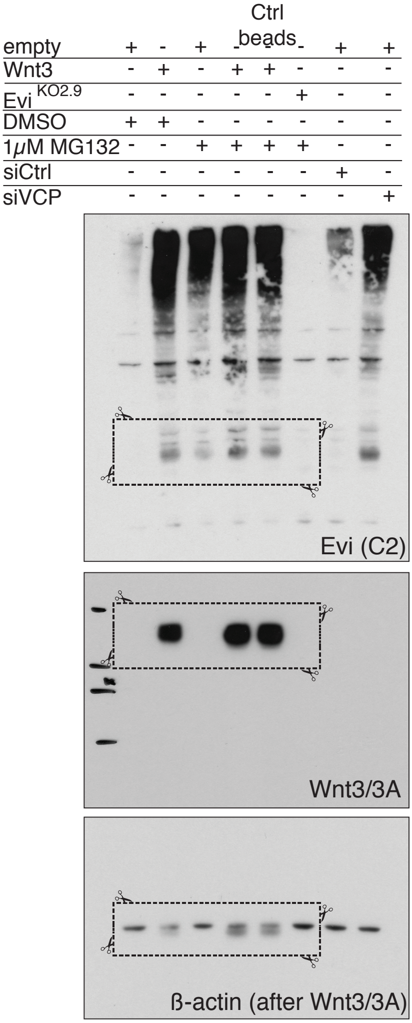

Fig3 D

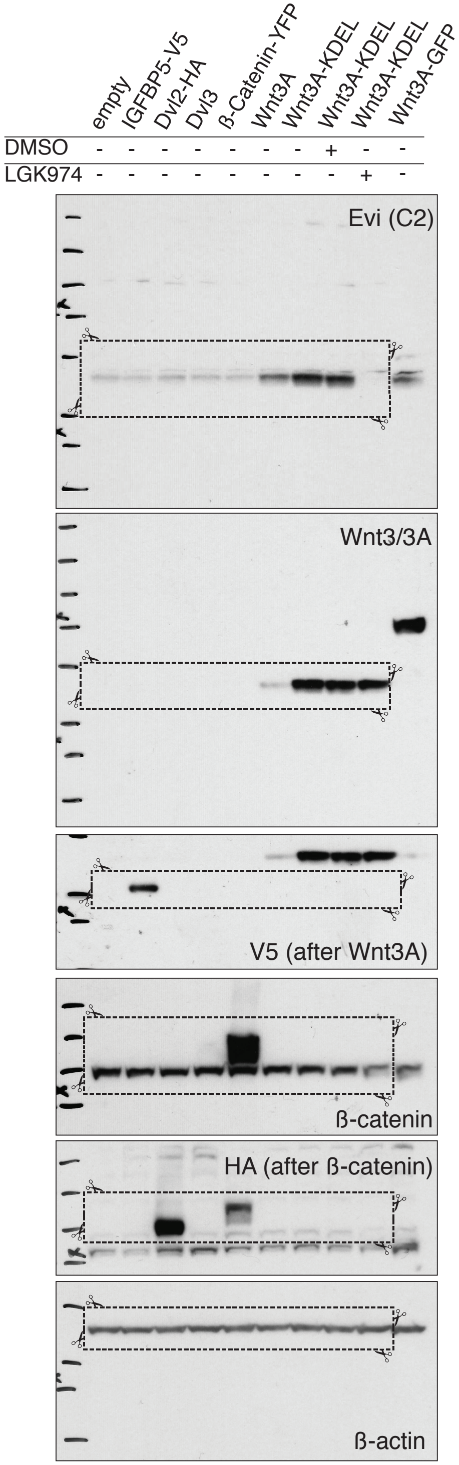
empty
IGFBP5-V5
Dvl2-HA
Dvl3
ß-Catenin-YFP
Wnt3A
Wnt3A-KDEL
Wnt3A-KDEL
Wnt3A-KDEL
Wnt3A-GFP
DMSO - - - - - - - + - -
LGK974 - - - - - - - - + -
Evi (C2)
Wnt3/3A
V5 (after Wnt3A)
ß-catenin
HA (after ß-catenin)
ß-actin
