# Supplementary material for: ERAD‐dependent control of the Wnt secretory factor Evi
Source: EMBO J. 2018 Jan 29;37(4):e97311. doi: 10.15252/embj.201797311 (PMC5813261; doi:10.15252/embj.201797311)
Supplement: Supplementary file 9 — Source Data for Figure 5 [file EMBJ-37-e97311-s007.pdf]

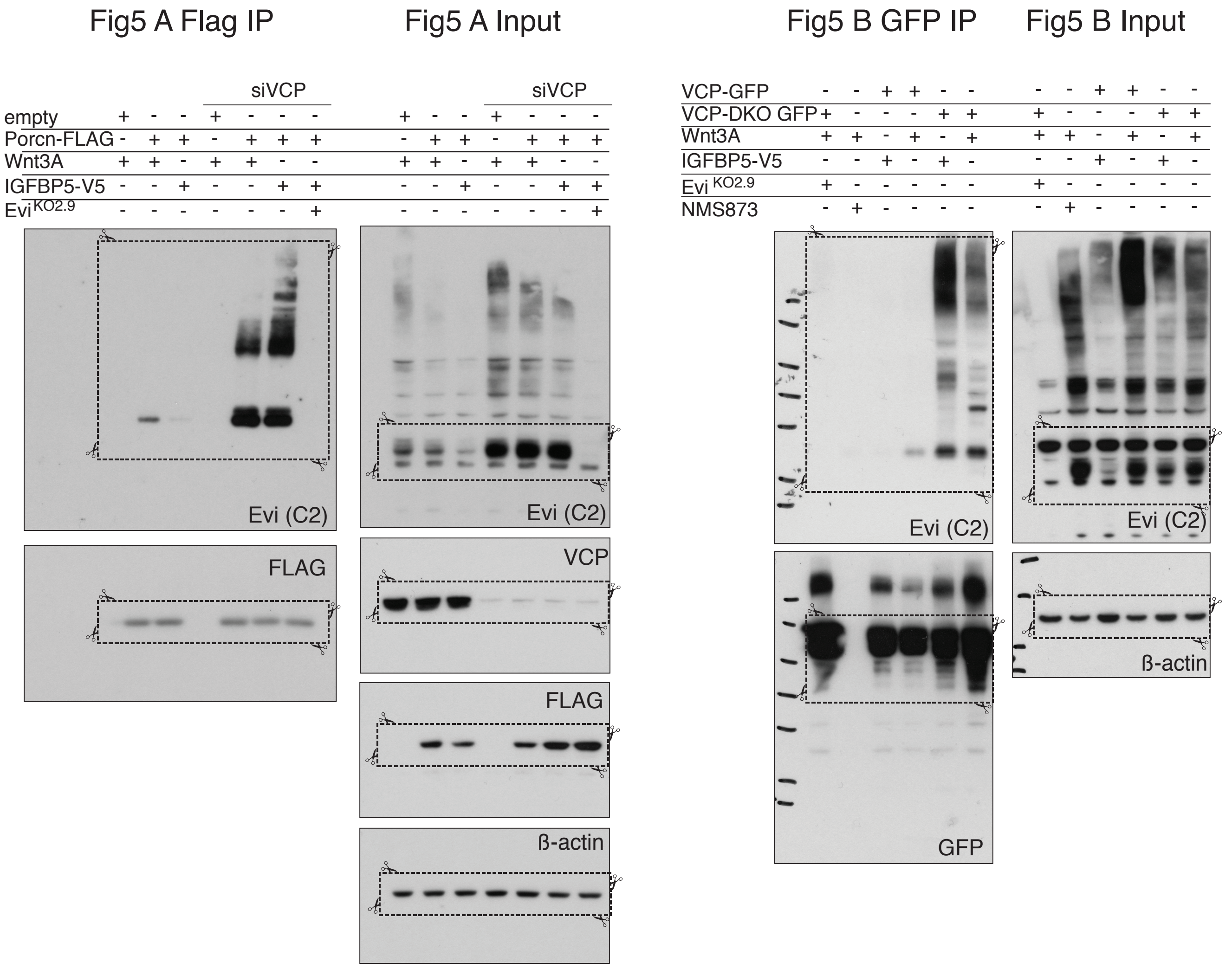
Fig5 A Flag IP
Fig5 A Input
Fig5 B GFP IP
Fig5 B Input
siVCP
empty
Porcn-FLAG
Wnt3A
IGFBP5-V5
Evi KO2.9
VCP-GFP
VCP-DKO GFP
NMS873
Evi (C2)
FLAG
VCP
ß-actin
GFP

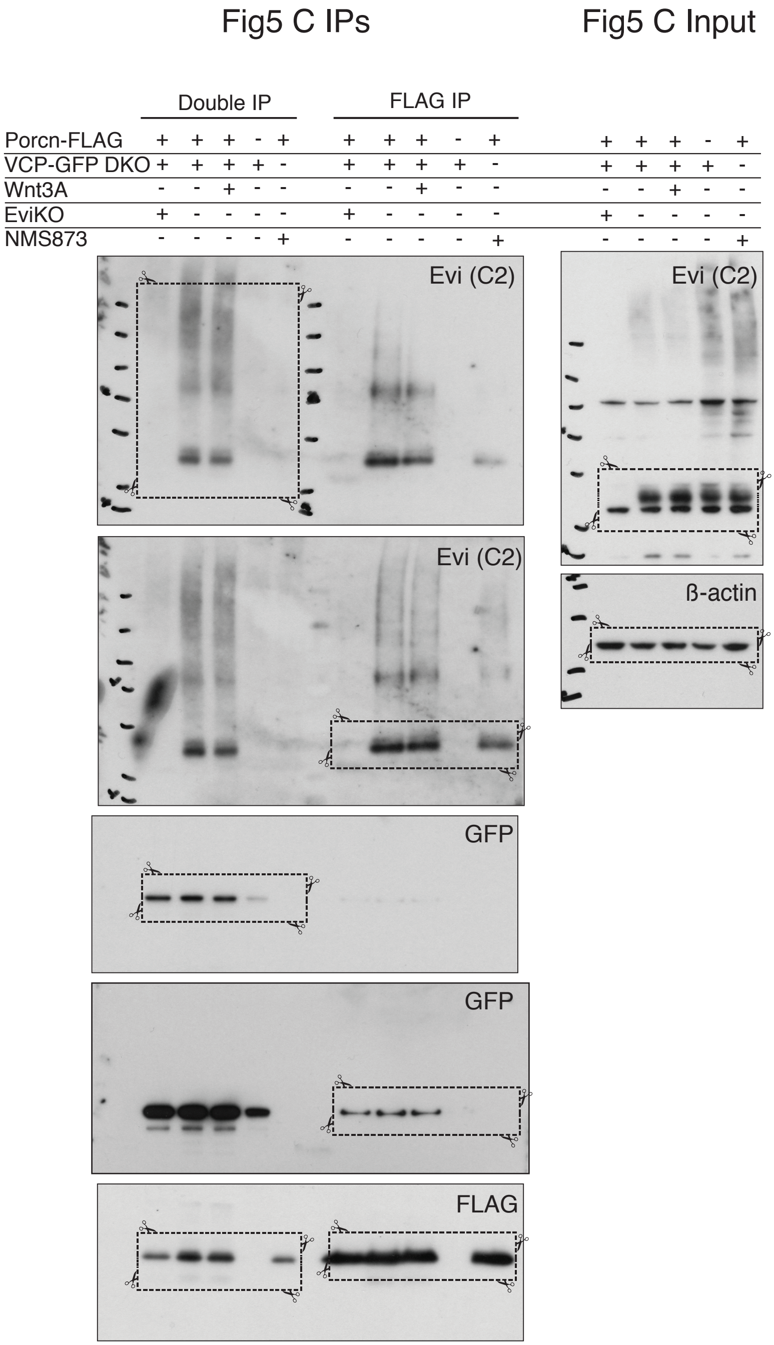

Fig5 C IPs
Fig5 C Input
Double IP
FLAG IP
Porcn-FLAG
VCP-GFP DKO
Wnt3A
EviKO
NMS873
Evi (C2)
Evi (C2)
GFP
GFP
FLAG
Evi (C2)
ß-actin
